# SOCRATES-CoMix: a platform for timely and open-source contact mixing data during and in between COVID-19 surges and interventions in over 20 European countries

**DOI:** 10.1186/s12916-021-02133-y

**Published:** 2021-09-29

**Authors:** Frederik Verelst, Lisa Hermans, Sarah Vercruysse, Amy Gimma, Pietro Coletti, Jantien A. Backer, Kerry L. M. Wong, James Wambua, Kevin van Zandvoort, Lander Willem, Laurens Bogaardt, Christel Faes, Christopher I. Jarvis, Jacco Wallinga, W. John Edmunds, Philippe Beutels, Niel Hens

**Affiliations:** 1 Centre for Health Economics Research and Modelling Infectious Diseases, Vaccine and Infectious Disease Institute, University of Antwerp, Antwerp, Belgium; 2 Data Science Institute and I-BioStat, Hasselt University, Hasselt, Belgium; 3 London School of Hygiene and Tropical Medicine, London, UK; 4 Centre for Infectious Disease Control, National Institute for Public Health and the Environment, Bilthoven, The Netherlands; 5 Dept Biomedical Data Sciences, Leiden University Medical Center, Leiden, The Netherlands; 6 School of Public Health and Community Medicine, The University of New South Wales, Sydney, Australia

**Keywords:** Social contact behaviour, Mixing patterns, Contact data, Mathematical modelling, SARS-CoV-2, COVID-19, Europe

## Abstract

**Background:**

SARS-CoV-2 dynamics are driven by human behaviour. Social contact data are of utmost importance in the context of transmission models of close-contact infections.

**Methods:**

Using online representative panels of adults reporting on their own behaviour as well as parents reporting on the behaviour of one of their children, we collect contact mixing (CoMix) behaviour in various phases of the COVID-19 pandemic in over 20 European countries.

We provide these timely, repeated observations using an online platform: SOCRATES-CoMix. In addition to providing cleaned datasets to researchers, the platform allows users to extract contact matrices that can be stratified by age, type of day, intensity of the contact and gender. These observations provide insights on the relative impact of recommended or imposed social distance measures on contacts and can inform mathematical models on epidemic spread.

**Conclusion:**

These data provide essential information for policymakers to balance non-pharmaceutical interventions, economic activity, mental health and wellbeing, during vaccine rollout.

**Supplementary Information:**

The online version contains supplementary material available at 10.1186/s12916-021-02133-y.

## Background

### Modelling a pandemic shaped by human behaviour

From the outbreak in December 2019 onwards, SARS-CoV-2 dynamics have been shaped by human behaviour [1]. For this reason, policymakers’ responses have been largely centred around social distancing measures to limit the burden of COVID-19 and to prevent healthcare systems from collapsing [2, 3]. Such measures—aimed at reducing the effective contact rate in society—will likely remain part of policymakers’ strategy until a substantial proportion of the population has been successfully vaccinated.

The importance of including social contact information in transmission models for close-contact infectious pathogens has been widely acknowledged in the literature, with the ‘social contact hypothesis’ [4] and the POLYMOD study [5] marking important milestones in the development and parameterization of such models. Over the past decades, social contact data have been increasingly used and collected in the context of transmission models of close-contact infections. A 2019 systematic review that retrieved 64 social contact studies reported common traits in terms of number of daily face-to-face conversational contacted persons (typically around 10 to 20) and general age-dependencies despite a variety of study designs [6].

Social contact pattern data have been indispensable for modelling SARS-CoV-2 transmission [7, 8]. Indeed, a number of SARS-CoV-2 modelling studies were capable of accurately and consistently predicting a variety of epidemiological parameters by relying on social contact data [9, 10]. Comparing reproduction numbers estimated from seroprevalence and virologic data to reproduction numbers estimated from social contact data in England, Davies et al. further validated the use of social contact data in the context of SARS-CoV-2 modelling [11]. Yet, adequate parameterization of such models requires country-specific social contact data collected under different policy interventions (e.g. lockdown versus no lockdown) and at different stages of the pandemic (e.g. in-between surges and during various ascending and descending stages of the pandemic).

## Construction and content

### CoMix: measuring behavioural change during the COVID-19 pandemic

CoMix is a longitudinal, multi-country social contact survey in representative panels of individuals in terms of age, gender, region of residence and—for most countries—either socio-economic status, occupation or educational attainment. The CoMix study started in March 2020, with survey data first being collected in the United Kingdom (UK), Belgium, and the Netherlands. It was set up to monitor awareness and behavioural changes during the pandemic. Each wave, panel members are invited to fill out the CoMix survey. On the survey day, participants retrospectively report all social contacts made from 5 am on the day preceding the survey up to 5 am on the day of the survey. A contact is defined as an in-person conversation of at least a few words or a skin-contact [9]. For every first wave, the target quota is set at 1500 participants, while a drop-out rate of 5 to 10% is allowed for every subsequent wave (except for some UK panels that were replenished with newly recruited participants and had a higher quota of 2500 later in the survey). When a significant proportion of the panel is lost to follow-up—after sending three reminder invitations—additional panel members are recruited up to the point where the sample matches the target quota. A CoMix wave refers to one period of survey data collection, running from the point when the invitations are sent up to the point when the quota are met and the survey is closed. We refer to the works by Jarvis et al. and Coletti et al. for further methodological details [7, 9]. Apart from participants’ social contacts, the survey also records individuals’ risk perceptions, such as the perceived severity of COVID-19, perceived susceptibility to COVID-19 and the perceived effectiveness of social distancing measures. Due to its longitudinal nature, the survey is particularly suited to quantify how changes in non-pharmaceutical interventions (NPIs) and changes in perception influence NPI compliance and social contact behaviour over time [12–15]. While most data is collected on behaviour in adults, a proportion of the respondents report contacts on behalf of their children. This provides crucial information about social mixing behaviour in children (and adults) when circumstances change (e.g. schools open versus closed) [8].

**Fig. 1:**
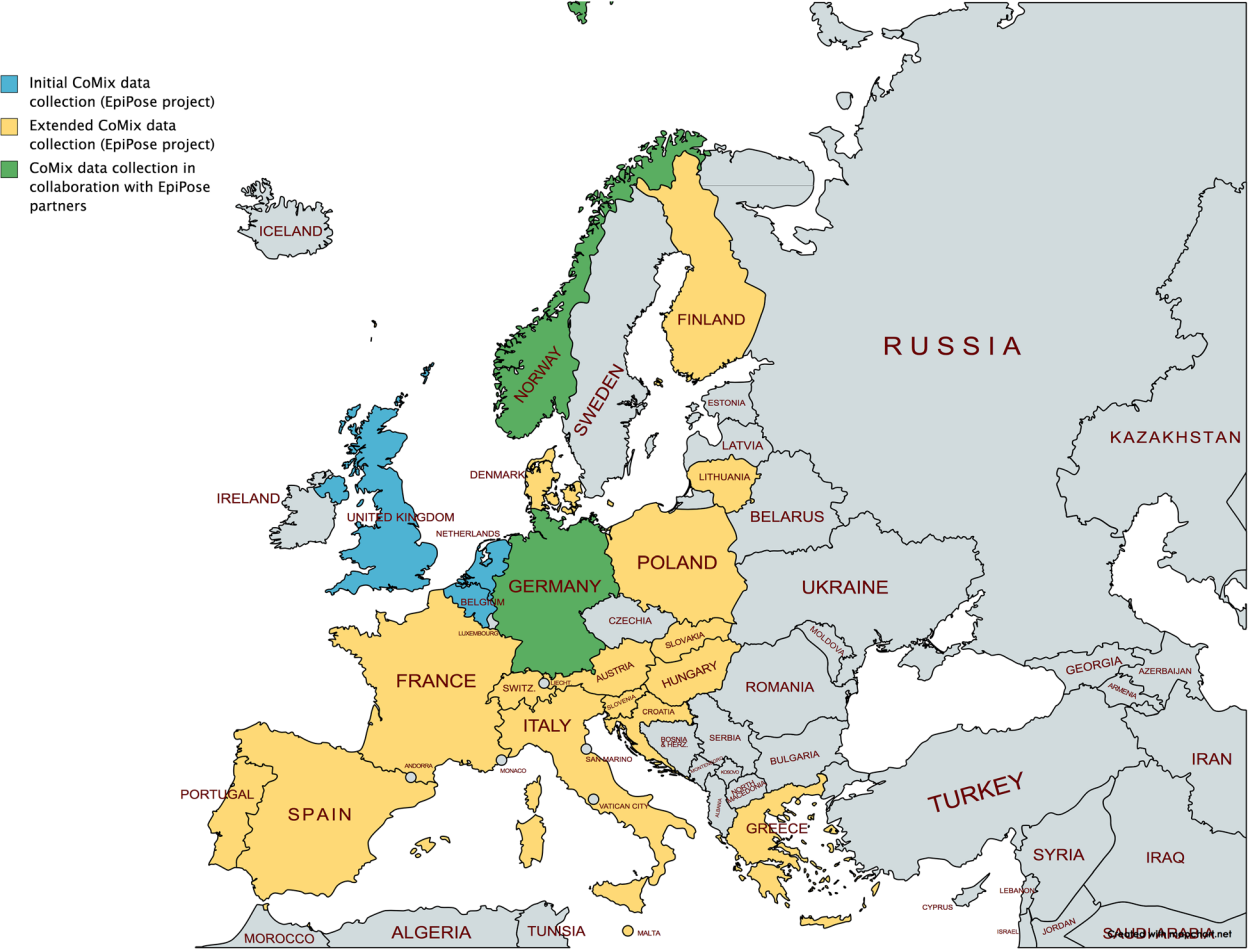
Map of Europe showing the geographical spread of CoMix and CoMix-like data collection

**Fig. 2:**
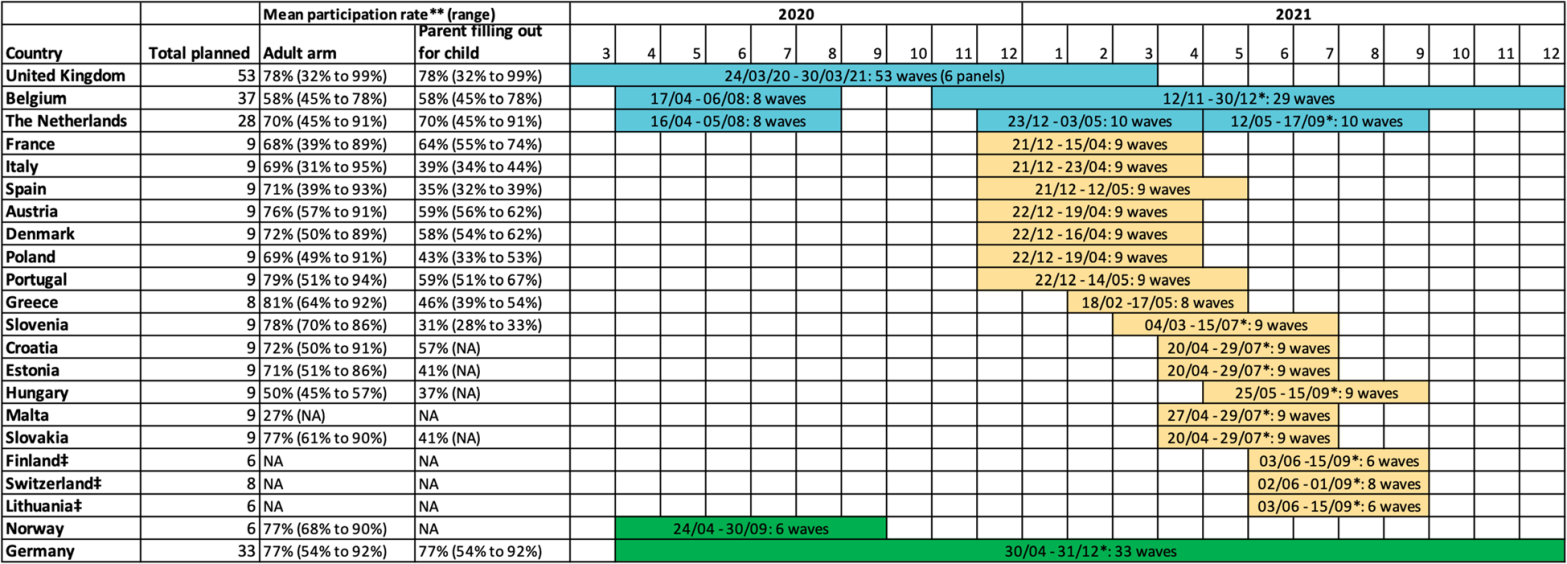
Overview of completed and scheduled CoMix data collection in Europe, as of 1 July 2021. Data collection in initial CoMix countries (as part of the EpiPose project) is depicted in blue, while extended CoMix data collection and data collection in collaboration with EpiPose partners are depicted in yellow and green, respectively. Colours correspond to the colours used in the map in Fig. 1. NA: Not applicable due to sample statistics not yet being available. * Estimated number or estimated timing. ** The participation rate is defined as the number of participants that completed the entire survey relative to the number of participants that opened the survey link. ‡ Due to data management issues during the initial data collection phase, parts of the data for Finland, Switzerland and Lithuania in Q1 2021 was removed by Ipsos due to quality concerns. As a result of a limited size of valid CoMix data collected for the Q1 2021 period, additional data is now being collected from June 2021 onwards

In October 2020, several European countries were faced with a surge in COVID-19 cases and had to resort to a second lockdown. Given the diverse range of policy measures in place across Europe [16]—and the central role of social contact data in the parameterization of infectious disease models—the CoMix study was extended to another 17 European countries. In addition, we invited research teams in Norway and Germany (COVIMOD study) that adapted the original CoMix survey to join the initiative in order to set up a collaborative network [17, 18]. The map in Fig. 1 shows the European countries that have been collecting social contact data within the context of—or similar to—the CoMix study. Figure 2 depicts an overview of the available and planned survey waves for all countries. Sample characteristics can be found in more detail in Additional file 1: Tables S1 to S19. We refer to the work by Coletti et al. [9] for sample characteristics for waves 1 to 8 in Belgium.

The CoMix data proved valuable to quantify the impact of social distancing measures over the course of the COVID-19 epidemic in the UK, Belgium and the Netherlands [7, 9, 13, 14, 19], and results are in line with other ongoing studies into social contact patterns with a different study population [19]. The extension of the CoMix study thus provides the opportunity to evaluate policies more accurately within and across a further 17 European countries.

### An open-source platform to extract SOcial Contact RATES (SOCRATES) from over 20 European countries

**Fig. 3:**
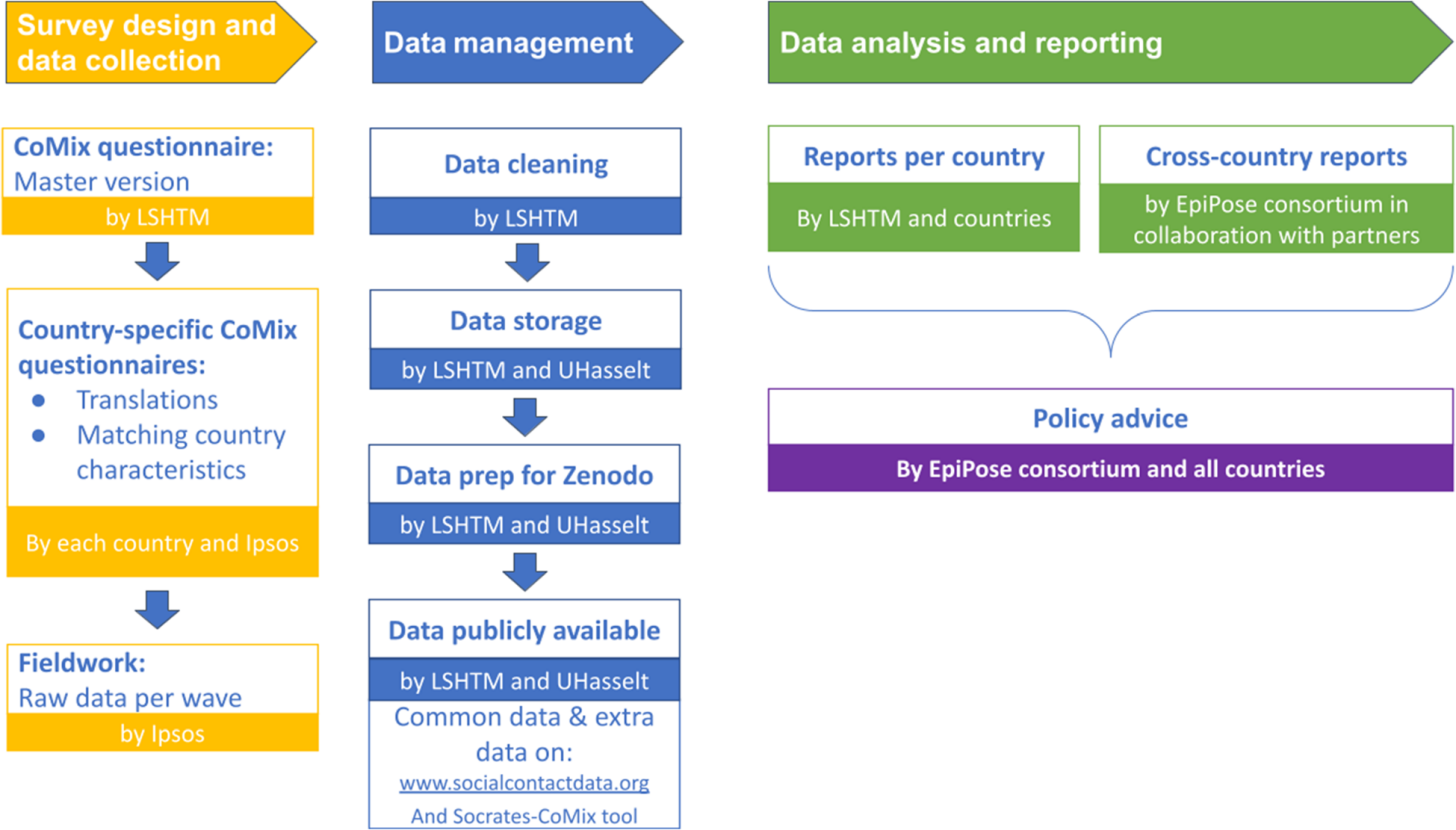
Schematic overview of the different steps in the CoMix study. The figure reflects the typical data flow for most European countries, yet deviations from this scheme are present in some. Abbreviations: LSHTM, London School of Hygiene and Tropical Medicine; UHasselt, Hasselt University; EpiPose, Epidemic intelligence to minimize COVID-19’s public health, social and economic impact. Ipsos is a commercial market research company

The typical CoMix data flow is reflected in Fig. 3, yet deviations from this scheme are present. The data flow starts from the ‘master’ version of the CoMix questionnaire that was developed and implemented early in the pandemic in the UK (24 March 2020). In collaboration with local partners in each country, the questionnaire is adapted to countries’ circumstances and languages, after which the fieldwork is implemented by a market research company. The CoMix data are cleaned and validated according to a data management protocol, the details and code which can be found on a GitHub repository [20]. After data cleaning, the data is stored and prepared for sharing in the public Zenodo-based repository (accessible via: www.socialcontactdata.org/data) as well as on the CoMix-Socrates tool. Furthermore, we invited partners performing a CoMix-like survey (Fig. 1) to also store their data on the public repository. CoMix data are analysed to gain insights at the national and international level which are converted into advice for health policymakers. The CoMix study protocols and questionnaires were approved—or waivers were obtained—by local ethical committees, the details of which can be found in Additional file 2: Table S20.

## Utility and discussion

### Usefulness and limitations of the SOCRATES-CoMix platform

**Fig. 4:**
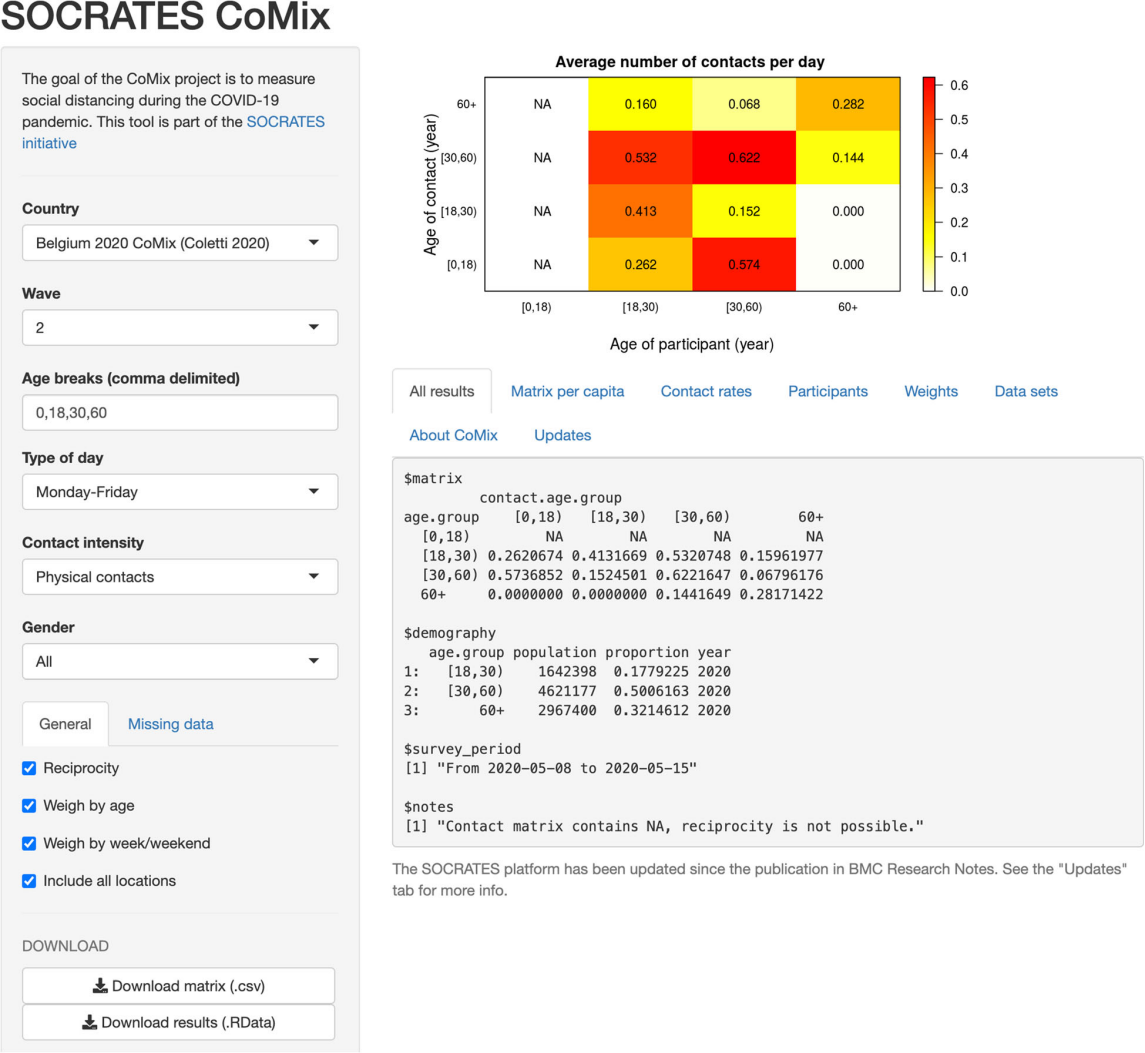
A print screen of the SOCRATES-CoMix tool. This specific example shows a social contact matrix using data collected in wave 2 of the Belgian CoMix study with four age classes, for weekdays and physical contacts only

As described by Willem et al. [3], the SOCRATES tool allows users to extract contact matrices and contact rates by country and survey wave. While the initial tool provides contact rates from a variety of contact studies by country and year, the newly developed SOCRATES-CoMix tool focuses on contact rates collected during the SARS-CoV-2 pandemic. The SOCRATES-CoMix tool allows stratification by age (user-picked age groups), type of day (week versus weekend), intensity of the contact (physical versus non-physical) and gender (see Fig. 4). Other features such as weighing by age and handling of missing data provides end-users the opportunity to match the social contact data extracted to their model requirements. We refer to the work of Willem et al. for further methodological details on how these data are summarised [3]. The platform is updated on a regular basis, adding social contact data from additional waves as they become available. The SOCRATES-CoMix tool can be found here: http://www.socialcontactdata.org/socrates-comix/.

The SOCRATES platform brings together social contact data from over 20 European countries collected at different points in time throughout the SARS-CoV-2 pandemic. Furthermore, the SOCRATES tool provides timely, cleaned and protracted social contact rates that can directly be integrated into mathematical models, while allowing for easy and quick data stratification, for instance by age or type of day, by use of a drop-down selection menu (Fig. 4). The platform also directs researchers to the public datasets on Zenodo repositories. CoMix data will continue to be of utmost importance in assessing the future course of the SARS-CoV-2 pandemic and to design effective public health policies. For example, in light of optimal COVID-19 vaccine roll-out, testing strategies and gradually less stringent NPIs [12].

Nevertheless, the CoMix data and the SOCRATES-CoMix tool have limitations. That is, the CoMix data are being collected in an online environment such that participants can only take part when they have access to a digital device, e.g. a personal computer, laptop, tablet or smartphone, and an internet connection. That means that CoMix data for older age groups are likely more prone to selection bias [21]. Selection bias, nevertheless, remains a concern for other age groups as well. In addition, self-completed surveys during a pandemic might be prone to social desirability bias, given mandatory social distancing measures and policymakers explicitly relying on the public’s social responsibility to protect others. However, social desirability bias may be minimal in view of the anonymous data entry, without direct contact with an interviewer. Lastly, due to respondents retrospectively reporting social contacts, the CoMix data may be prone to recall bias. Yet, given that participants are reporting all contacts made between 5 am the day preceding the survey and 5 am of the day of the survey, we believe the scope for recall bias is very limited.

## Conclusion

Epidemic modelling can be enhanced with data describing contact patterns of individuals. In order to understand, model and respond to the COVID-19 pandemic in a timely manner, there was a great need for social contact data. As the virus does not stay within a country’s borders, the data collection has been set up in many European countries.

Scientific and policy-related insights can be drawn for each country, but even more, cross-country analyses are feasible. Combined with mathematical models, these data provide insights for policymakers, balancing non-pharmaceutical interventions, economic activity, mental health and wellbeing, also during the vaccine rollout.

## Supplementary Information

**Additional file 1:** CoMix sample characteristics by country and survey wave. Additional file 1 provides sample characteristics of CoMix waves that were already collected and of which the data has been delivered. Sample characteristics may deviate from the ones reported in other CoMix studies as a result of data cleaning or other post-collection corrections. CoMix data collection in Finland, Switzerland and Lithuania was postponed to summer 2021 due to an issue in the data collection. Hence, no sample characteristics are reported for these three countries. **Tables S1-S19.** - CoMix sample characteristics for the United Kingdom, Belgium, The Netherlands, France, Italy, Spain, Austria, Denmark, Poland, Portugal, Greece, Slovenia, Croatia, Estonia, Hungary, Malta, Slovakia, Norway and Germany.

**Additional file 2:** Country-level ethics details. Additional file 2 provides country-level details of the ethical approvals, or waivers, for the CoMix study protocol and questionnaires. **Table S20.** - Country-level details of the ethical approvals, or waivers, for the CoMix study protocol and questionnaires.

## Data Availability

The datasets generated and/or analysed during the current study are available in the Zenodo-based repository, www.socialcontactdata.org/data, as well as on the CoMix-Socrates tool, http://www.socialcontactdata.org/socrates-comix/.

## Abbreviations

CoMix: Contact mixing
UK: United Kingdom
NPI: Non-pharmaceutical intervention
SOCRATES: Social contact rates
NA: Not applicable
LSHTM: London School of Hygiene and Tropical Medicine
UHasselt: Hasselt University
EpiPose: Epidemic intelligence to minimize COVID-19’s public health, social and economic impact
