# Supplementary material for: SOCRATES-CoMix: a platform for timely and open-source contact mixing data during and in between COVID-19 surges and interventions in over 20 European countries
Source: BMC Med. 2021 Sep 29;19:254. doi: 10.1186/s12916-021-02133-y (PMC8478607; doi:10.1186/s12916-021-02133-y)
Supplement: Supplementary file 1 — Additional file 1: CoMix sample characteristics by country and survey wave. Additional file 1 provides sample characteristics of CoMix waves that were already collected and of which the data has been delivered. Sample characteristics may deviate from the ones reported in other CoMix studies as a result of data cleaning or other post-collection corrections. CoMix data collection in Finland, Switzerland and Lithuania was postponed to summer 2021 due to an issue in the data collection. Hence, no sample characteristics are reported for these three countries. Tables S1-S19. - CoMix sample characteristics for the United Kingdom, Belgium, The Netherlands, France, Italy, Spain, Austria, Denmark, Poland, Portugal, Greece, Slovenia, Croatia, Estonia, Hungary, Malta, Slovakia, Norway and Germany. [file 12916_2021_2133_MOESM1_ESM.pdf]

Additional file 1 for: “SOCRATES-CoMix: A platform for timely and open-source contact mixing data during and in between COVID-19 surges and interventions in over 20 European countries”

Frederik Verelst[1], Lisa Hermans[2*], Sarah Vercruysse[2], Amy Gimma[3], Pietro Coletti[2], Jantien A Backer[4], Kerry LM Wong[3], James Wambua[2], Kevin van Zandvoort[3], Lander Willem[1], Laurens Bogaardt[4], Christel Faes[2], Christopher I Jarvis[3], Jacco Wallinga[4,5], W John Edmunds[3], Philippe Beutels[1,6], Niel Hens[1,2]

1 Centre for Health Economics Research and Modelling Infectious Diseases, Vaccine and Infectious Disease Institute, University of Antwerp, Antwerp, Belgium

2 Data Science Institute and I-BioStat, Hasselt University, Hasselt, Belgium

3 London School of Hygiene and Tropical Medicine, London, UK

4 Centre for Infectious Disease Control, National Institute for Public Health and the Environment, Bilthoven, the Netherlands

5 Dept Biomedical Data Sciences, Leiden University Medical Center, Leiden, the Netherlands

6 School of Public Health and Community Medicine, The University of New South Wales, Sydney, Australia

*Corresponding author: Lisa Hermans, lisa.hermans@uhasselt.be

Contents

1 Additional file 1 : CoMix sample characteristics by country and survey wave 3

1.1 United Kingdom 4

1.2 Belgium 14

1.3 The Netherlands 16

1.4 France 20

1.5 Italy 21

1.6 Spain 22

1.7 Austria 23

1.8 Denmark 24

1.9 Poland 25

1.10 Portugal 26

1.11 Greece 27

1.12 Slovenia 28

1.13 Croatia 29

1.14 Estonia 30

1.15 Hungary 31

1.16 Malta 32

1.17 Slovakia 33

1.18 Norway 34

1.19 Germany 35

1 Additional file 1 : CoMix sample characteristics by country and survey wave

This additional file provides sample characteristics of CoMix waves that were already collected and of which the data has been delivered. Sample characteristics may deviate from the ones reported in other CoMix studies as a result of data cleaning or other post-collection corrections. CoMix data collection in Finland, Switzerland and Lithuania was postponed to summer 2021 due to an issue in the data collection. Hence, no sample characteristics are reported for these three countries.

1.1 United Kingdom

Table S1. CoMix sample characteristics for the United Kingdom.

Characteristic	Wave A 1	Wave A 2	Wave A 3	Wave A 4	Wave A 5	Wave A 6	Wave A 7	Wave C 7	Wave A 8	Wave C 8
**All**	1816	1560	1326	1326	1144	1127	1435	750	1146	507
**Age**										
0-4	NA	NA	NA	NA	NA	NA	NA	104 (13.9%)	NA	92 (18.1%)
5-11	NA	NA	NA	NA	NA	NA	NA	264 (35.2%)	NA	180 (35.5%)
12-17	NA	NA	NA	NA	NA	NA	NA	371 (49.5%)	NA	223 (44%)
18-29	275 (15.1%)	200 (12.8%)	145 (10.9%)	129 (9.7%)	128 (11.2%)	76 (6.7%)	137 (9.5%)	NA	132 (11.5%)	NA
30-39	334 (18.4%)	233 (14.9%)	215 (16.2%)	169 (12.7%)	179 (15.6%)	137 (12.2%)	248 (17.3%)	NA	130 (11.3%)	NA
40-49	297 (16.4%)	306 (19.6%)	210 (15.8%)	258 (19.5%)	210 (18.4%)	217 (19.3%)	239 (16.7%)	NA	214 (18.7%)	NA
50-59	295 (16.2%)	322 (20.6%)	249 (18.8%)	300 (22.6%)	194 (17%)	266 (23.6%)	245 (17.1%)	NA	249 (21.7%)	NA
60-69	422 (23.2%)	248 (15.9%)	354 (26.7%)	233 (17.6%)	306 (26.7%)	210 (18.6%)	384 (26.8%)	NA	205 (17.9%)	NA
70+	193 (10.6%)	251 (16.1%)	153 (11.5%)	237 (17.9%)	127 (11.1%)	221 (19.6%)	182 (12.7%)	NA	216 (18.8%)	NA
Unknown age	NA	NA	NA	NA	NA	NA	NA	11 (1.5%)	NA	12 (2.4%)
**Gender**										
Female	878 (48.3%)	851 (54.6%)	642 (48.4%)	723 (54.5%)	544 (47.6%)	618 (54.8%)	692 (48.2%)	352 (46.9%)	634 (55.3%)	247 (48.7%)
Male	932 (51.3%)	697 (44.7%)	680 (51.3%)	599 (45.2%)	598 (52.3%)	504 (44.7%)	739 (51.5%)	396 (52.8%)	506 (44.2%)	256 (50.5%)
Other	6 (0.3%)	12 (0.8%)	4 (0.3%)	4 (0.3%)	2 (0.2%)	5 (0.4%)	4 (0.3%)	2 (0.3%)	6 (0.5%)	4 (0.8%)
**Day**										
Sunday	5 (0.3%)	162 (10.4%)	11 (0.8%)	34 (2.6%)	49 (4.3%)	29 (2.6%)	29 (2%)	36 (4.8%)	NA	58 (11.4%)
Monday	8 (0.4%)	314 (20.1%)	37 (2.8%)	45 (3.4%)	17 (1.5%)	151 (13.4%)	20 (1.4%)	37 (4.9%)	90 (7.9%)	41 (8.1%)
Tuesday	558 (30.7%)	170 (10.9%)	310 (23.4%)	112 (8.4%)	NA	24 (2.1%)	835 (58.2%)	82 (10.9%)	63 (5.5%)	21 (4.1%)
Wednesday	1025 (56.4%)	95 (6.1%)	756 (57%)	27 (2%)	NA	86 (7.6%)	208 (14.5%)	127 (16.9%)	34 (3%)	21 (4.1%)
Thursday	162 (8.9%)	256 (16.4%)	111 (8.4%)	383 (28.9%)	87 (7.6%)	89 (7.9%)	244 (17%)	14 (1.9%)	216 (18.8%)	80 (15.8%)
Friday	36 (2%)	383 (24.6%)	72 (5.4%)	647 (48.8%)	869 (76%)	695 (61.7%)	60 (4.2%)	21 (2.8%)	666 (58.1%)	202 (39.8%)
Saturday	22 (1.2%)	180 (11.5%)	29 (2.2%)	78 (5.9%)	122 (10.7%)	53 (4.7%)	39 (2.7%)	433 (57.7%)	77 (6.7%)	84 (16.6%)
**Data collection**										
First day	24 Mar 20	02 Apr 20	07 Apr 20	16 Apr 20	23 Apr 20	30 Apr 20	05 May 20	09 May 20	14 May 20	15 May 20
Last day	01 Apr 20	10 Apr 20	15 Apr 20	24 Apr 20	27 Apr 20	07 May 20	12 May 20	18 May 20	21 May 20	25 May 20
**Household size**										
1	286 (15.7%)	289 (18.5%)	272 (20.5%)	291 (21.9%)	244 (21.3%)	259 (23%)	301 (21%)	NA	259 (22.6%)	NA
2	596 (32.8%)	521 (33.4%)	486 (36.7%)	479 (36.1%)	432 (37.8%)	426 (37.8%)	581 (40.5%)	23 (3.1%)	439 (38.3%)	20 (3.9%)
3-5	867 (47.7%)	704 (45.1%)	533 (40.2%)	524 (39.5%)	441 (38.5%)	423 (37.5%)	521 (36.3%)	634 (84.5%)	432 (37.7%)	445 (87.8%)
6+	67 (3.7%)	46 (2.9%)	35 (2.6%)	32 (2.4%)	27 (2.4%)	19 (1.7%)	32 (2.2%)	93 (12.4%)	16 (1.4%)	42 (8.3%)
**Panel**	A	B	A	B	A	B	A	C	B	D

Note: Wave A refers to CoMix surveys collecting behavioural data for adults whereas Wave C refers to CoMix surveys collecting behavioural data from adults responding on behalf of a randomly selected child.

Table S1 (continued) CoMix sample characteristics for the United Kingdom.

Characteristic	Wave A 9	Wave C 9	Wave A 10	Wave C 10	Wave A 11	Wave C 11	Wave A 12	Wave C 12	Wave A 13	Wave C 13
**All**	1415	512	1082	359	1339	511	996	305	1335	509
**Age**										
0-4	NA	63 (12.3%)	NA	61 (17%)	NA	58 (11.4%)	NA	44 (14.4%)	NA	65 (12.8%)
5-11	NA	166 (32.4%)	NA	125 (34.8%)	NA	164 (32.1%)	NA	107 (35.1%)	NA	167 (32.8%)
12-17	NA	259 (50.6%)	NA	155 (43.2%)	NA	260 (50.9%)	NA	139 (45.6%)	NA	256 (50.3%)
18-29	131 (9.3%)	NA	100 (9.2%)	NA	114 (8.5%)	NA	86 (8.6%)	NA	117 (8.8%)	NA
30-39	229 (16.2%)	NA	131 (12.1%)	NA	221 (16.5%)	NA	105 (10.5%)	NA	215 (16.1%)	NA
40-49	244 (17.2%)	NA	207 (19.1%)	NA	224 (16.7%)	NA	203 (20.4%)	NA	218 (16.3%)	NA
50-59	254 (18%)	NA	243 (22.5%)	NA	240 (17.9%)	NA	243 (24.4%)	NA	241 (18.1%)	NA
60-69	382 (27%)	NA	202 (18.7%)	NA	366 (27.3%)	NA	184 (18.5%)	NA	368 (27.6%)	NA
70+	175 (12.4%)	NA	199 (18.4%)	NA	174 (13%)	NA	175 (17.6%)	NA	176 (13.2%)	NA
Unknown age	NA	24 (4.7%)	NA	18 (5%)	NA	29 (5.7%)	NA	15 (4.9%)	NA	21 (4.1%)
**Gender**										
Female	691 (48.8%)	234 (45.7%)	599 (55.4%)	171 (47.6%)	643 (48%)	238 (46.6%)	538 (54%)	141 (46.2%)	637 (47.7%)	246 (48.3%)
Male	718 (50.7%)	277 (54.1%)	479 (44.3%)	187 (52.1%)	693 (51.8%)	270 (52.8%)	455 (45.7%)	163 (53.4%)	693 (51.9%)	263 (51.7%)
Other	6 (0.4%)	1 (0.2%)	4 (0.4%)	1 (0.3%)	3 (0.2%)	3 (0.6%)	3 (0.3%)	1 (0.3%)	5 (0.4%)	NA
**Day**										
Sunday	28 (2%)	70 (13.7%)	45 (4.2%)	25 (7%)	32 (2.4%)	63 (12.3%)	28 (2.8%)	18 (5.9%)	18 (1.3%)	10 (2%)
Monday	81 (5.7%)	33 (6.4%)	20 (1.8%)	26 (7.2%)	35 (2.6%)	180 (35.2%)	11 (1.1%)	11 (3.6%)	92 (6.9%)	20 (3.9%)
Tuesday	40 (2.8%)	70 (13.7%)	94 (8.7%)	16 (4.5%)	20 (1.5%)	34 (6.7%)	NA	1 (0.3%)	41 (3.1%)	40 (7.9%)
Wednesday	35 (2.5%)	13 (2.5%)	27 (2.5%)	149 (41.5%)	75 (5.6%)	48 (9.4%)	34 (3.4%)	53 (17.4%)	5 (0.4%)	13 (2.6%)
Thursday	474 (33.5%)	2 (0.4%)	291 (26.9%)	25 (7%)	468 (35%)	1 (0.2%)	277 (27.8%)	89 (29.2%)	483 (36.2%)	182 (35.8%)
Friday	657 (46.4%)	NA	491 (45.4%)	81 (22.6%)	630 (47.1%)	14 (2.7%)	559 (56.1%)	105 (34.4%)	635 (47.6%)	214 (42%)
Saturday	100 (7.1%)	324 (63.3%)	114 (10.5%)	37 (10.3%)	79 (5.9%)	171 (33.5%)	87 (8.7%)	28 (9.2%)	61 (4.6%)	30 (5.9%)
**Data collection**										
First day	21 May 20	23 May 20	28 May 20	28 May 20	04 Jun 20	05 Jun 20	11 Jun 20	11 Jun 20	18 Jun 20	18 Jun 20
Last day	28 May 20	28 May 20	03 Jun 20	04 Jun 20	11 Jun 20	11 Jun 20	18 Jun 20	17 Jun 20	24 Jun 20	24 Jun 20
**Household size**										
1	298 (21.1%)	NA	257 (23.8%)	NA	283 (21.1%)	NA	243 (24.4%)	NA	295 (22.1%)	NA
2	566 (40%)	15 (2.9%)	429 (39.6%)	18 (5%)	571 (42.6%)	12 (2.3%)	397 (39.9%)	15 (4.9%)	566 (42.4%)	14 (2.8%)
3-5	528 (37.3%)	439 (85.7%)	378 (34.9%)	311 (86.6%)	465 (34.7%)	441 (86.3%)	340 (34.1%)	265 (86.9%)	448 (33.6%)	439 (86.2%)
6+	23 (1.6%)	58 (11.3%)	18 (1.7%)	30 (8.4%)	20 (1.5%)	58 (11.4%)	16 (1.6%)	25 (8.2%)	26 (1.9%)	56 (11%)
**Panel**	A	C	B	D	A	C	B	D	A	C

Note: Wave A refers to CoMix surveys collecting behavioural data for adults whereas Wave C refers to CoMix surveys collecting behavioural data from adults responding on behalf of a randomly selected child.

Table S1 (continued) CoMix sample characteristics for the United Kingdom.

Characteristic	Wave A 14	Wave C 14	Wave A 15	Wave C 15	Wave A 16	Wave C 16	Wave A 17	Wave A 18	Wave A 19	Wave C 19
**All**	958	250	1326	516	959	200	1068	958	1236	437
**Age**										
0-4	NA	37 (14.8%)	NA	66 (12.8%)	NA	33 (16.5%)	NA	NA	NA	49 (11.2%)
5-11	NA	82 (32.8%)	NA	162 (31.4%)	NA	67 (33.5%)	NA	NA	NA	137 (31.4%)
12-17	NA	115 (46%)	NA	267 (51.7%)	NA	89 (44.5%)	NA	NA	NA	234 (53.5%)
18-29	72 (7.5%)	NA	105 (7.9%)	NA	76 (7.9%)	NA	54 (5.1%)	79 (8.2%)	93 (7.5%)	NA
30-39	97 (10.1%)	NA	219 (16.5%)	NA	113 (11.8%)	NA	154 (14.4%)	114 (11.9%)	196 (15.9%)	NA
40-49	196 (20.5%)	NA	213 (16.1%)	NA	183 (19.1%)	NA	152 (14.2%)	185 (19.3%)	191 (15.5%)	NA
50-59	220 (23%)	NA	241 (18.2%)	NA	223 (23.3%)	NA	200 (18.7%)	220 (23%)	235 (19%)	NA
60-69	179 (18.7%)	NA	371 (28%)	NA	179 (18.7%)	NA	344 (32.2%)	170 (17.7%)	350 (28.3%)	NA
70+	194 (20.3%)	NA	177 (13.3%)	NA	185 (19.3%)	NA	164 (15.4%)	190 (19.8%)	171 (13.8%)	NA
Unknown age	NA	16 (6.4%)	NA	21 (4.1%)	NA	11 (5.5%)	NA	NA	NA	17 (3.9%)
**Gender**										
Female	503 (52.5%)	124 (49.6%)	638 (48.1%)	239 (46.3%)	523 (54.5%)	87 (43.5%)	509 (47.7%)	518 (54.1%)	596 (48.2%)	212 (48.5%)
Male	452 (47.2%)	125 (50%)	685 (51.7%)	277 (53.7%)	430 (44.8%)	113 (56.5%)	556 (52.1%)	434 (45.3%)	636 (51.5%)	224 (51.3%)
Other	3 (0.3%)	1 (0.4%)	3 (0.2%)	NA	6 (0.6%)	NA	3 (0.3%)	6 (0.6%)	4 (0.3%)	1 (0.2%)
**Day**										
Sunday	52 (5.4%)	14 (5.6%)	27 (2%)	19 (3.7%)	245 (25.5%)	10 (5%)	258 (24.2%)	46 (4.8%)	58 (4.7%)	10 (2.3%)
Monday	21 (2.2%)	30 (12%)	107 (8.1%)	47 (9.1%)	71 (7.4%)	19 (9.5%)	354 (33.1%)	234 (24.4%)	283 (22.9%)	195 (44.6%)
Tuesday	NA	6 (2.4%)	67 (5.1%)	21 (4.1%)	29 (3%)	NA	42 (3.9%)	256 (26.7%)	342 (27.7%)	103 (23.6%)
Wednesday	NA	43 (17.2%)	21 (1.6%)	14 (2.7%)	NA	1 (0.5%)	158 (14.8%)	58 (6.1%)	78 (6.3%)	53 (12.1%)
Thursday	293 (30.6%)	59 (23.6%)	447 (33.7%)	161 (31.2%)	251 (26.2%)	74 (37%)	109 (10.2%)	159 (16.6%)	32 (2.6%)	36 (8.2%)
Friday	510 (53.2%)	76 (30.4%)	586 (44.2%)	218 (42.2%)	183 (19.1%)	68 (34%)	11 (1%)	145 (15.1%)	315 (25.5%)	30 (6.9%)
Saturday	82 (8.6%)	22 (8.8%)	71 (5.4%)	36 (7%)	180 (18.8%)	28 (14%)	136 (12.7%)	60 (6.3%)	128 (10.4%)	10 (2.3%)
**Data collection**										
First day	25 Jun 20	25 Jun 20	02 Jul 20	02 Jul 20	09 Jul 20	09 Jul 20	16 Jul 20	23 Jul 20	31 Jul 20	03 Aug 20
Last day	29 Jun 20	01 Jul 20	08 Jul 20	09 Jul 20	14 Jul 20	16 Jul 20	23 Jul 20	29 Jul 20	07 Aug 20	09 Aug 20
**Household size**										
1	235 (24.5%)	NA	301 (22.7%)	NA	252 (26.3%)	NA	285 (26.7%)	252 (26.3%)	288 (23.3%)	NA
2	389 (40.6%)	11 (4.4%)	574 (43.3%)	16 (3.1%)	387 (40.4%)	9 (4.5%)	499 (46.7%)	382 (39.9%)	525 (42.5%)	15 (3.4%)
3-5	319 (33.3%)	218 (87.2%)	430 (32.4%)	439 (85.1%)	313 (32.6%)	175 (87.5%)	270 (25.3%)	311 (32.5%)	409 (33.1%)	375 (85.8%)
6+	15 (1.6%)	21 (8.4%)	21 (1.6%)	61 (11.8%)	7 (0.7%)	16 (8%)	14 (1.3%)	13 (1.4%)	14 (1.1%)	47 (10.8%)
**Panel**	B	D	A	C	B	D	A	B	A	C

Note: Wave A refers to CoMix surveys collecting behavioural data for adults whereas Wave C refers to CoMix surveys collecting behavioural data from adults responding on behalf of a randomly selected child.

Table S1 (continued) CoMix sample characteristics for the United Kingdom.

Characteristic	Wave A 20	Wave C 20	Wave A 21	Wave C 21	Wave A 22	Wave C 22	Wave A 23	Wave C 23	Wave A 24	Wave C 24
**All**	3039	759	3238	759	2483	557	2653	386	2066	516
**Age**										
0-4	NA	147 (19.4%)	NA	116 (15.3%)	NA	102 (18.3%)	NA	51 (13.2%)	NA	102 (19.8%)
5-11	NA	267 (35.2%)	NA	295 (38.9%)	NA	178 (32%)	NA	143 (37%)	NA	179 (34.7%)
12-17	NA	316 (41.6%)	NA	325 (42.8%)	NA	235 (42.2%)	NA	171 (44.3%)	NA	192 (37.2%)
18-29	513 (16.9%)	NA	554 (17.1%)	NA	351 (14.1%)	NA	368 (13.9%)	NA	258 (12.5%)	NA
30-39	526 (17.3%)	NA	552 (17%)	NA	385 (15.5%)	NA	400 (15.1%)	NA	282 (13.6%)	NA
40-49	536 (17.6%)	NA	500 (15.4%)	NA	390 (15.7%)	NA	412 (15.5%)	NA	339 (16.4%)	NA
50-59	603 (19.8%)	NA	630 (19.5%)	NA	472 (19%)	NA	545 (20.5%)	NA	404 (19.6%)	NA
60-69	575 (18.9%)	NA	564 (17.4%)	NA	561 (22.6%)	NA	520 (19.6%)	NA	499 (24.2%)	NA
70+	286 (9.4%)	NA	438 (13.5%)	NA	324 (13%)	NA	408 (15.4%)	NA	284 (13.7%)	NA
Unknown age	NA	29 (3.8%)	NA	23 (3%)	NA	42 (7.5%)	NA	21 (5.4%)	NA	43 (8.3%)
**Gender**										
Female	1600 (52.6%)	344 (45.3%)	1714 (52.9%)	368 (48.5%)	1320 (53.2%)	248 (44.5%)	1382 (52.1%)	193 (50%)	1079 (52.2%)	244 (47.3%)
Male	1427 (47%)	412 (54.3%)	1510 (46.6%)	388 (51.1%)	1160 (46.7%)	307 (55.1%)	1266 (47.7%)	191 (49.5%)	982 (47.5%)	270 (52.3%)
Other	12 (0.4%)	3 (0.4%)	14 (0.4%)	3 (0.4%)	3 (0.1%)	2 (0.4%)	5 (0.2%)	2 (0.5%)	5 (0.2%)	2 (0.4%)
**Day**										
Sunday	14 (0.5%)	3 (0.4%)	275 (8.5%)	71 (9.4%)	26 (1%)	10 (1.8%)	58 (2.2%)	7 (1.8%)	NA	NA
Monday	928 (30.5%)	549 (72.3%)	927 (28.6%)	211 (27.8%)	133 (5.4%)	42 (7.5%)	1357 (51.1%)	229 (59.3%)	671 (32.5%)	191 (37%)
Tuesday	602 (19.8%)	109 (14.4%)	315 (9.7%)	59 (7.8%)	316 (12.7%)	75 (13.5%)	195 (7.4%)	49 (12.7%)	122 (5.9%)	21 (4.1%)
Wednesday	749 (24.6%)	51 (6.7%)	21 (0.6%)	22 (2.9%)	1599 (64.4%)	307 (55.1%)	107 (4%)	22 (5.7%)	969 (46.9%)	134 (26%)
Thursday	212 (7%)	16 (2.1%)	14 (0.4%)	14 (1.8%)	275 (11.1%)	74 (13.3%)	314 (11.8%)	3 (0.8%)	285 (13.8%)	168 (32.6%)
Friday	473 (15.6%)	24 (3.2%)	1103 (34.1%)	211 (27.8%)	98 (3.9%)	36 (6.5%)	172 (6.5%)	31 (8%)	19 (0.9%)	2 (0.4%)
Saturday	61 (2%)	7 (0.9%)	583 (18%)	171 (22.5%)	36 (1.4%)	13 (2.3%)	450 (17%)	45 (11.7%)	NA	NA
**Data collection**										
First day	10 Aug 20	10 Aug 20	14 Aug 20	14 Aug 20	24 Aug 20	25 Aug 20	28 Aug 20	28 Aug 20	07 Sep 20	07 Sep 20
Last day	19 Aug 20	18 Aug 20	25 Aug 20	24 Aug 20	02 Sep 20	02 Sep 20	04 Sep 20	04 Sep 20	11 Sep 20	11 Sep 20
**Household size**										
1	673 (22.1%)	NA	772 (23.8%)	NA	590 (23.8%)	NA	681 (25.7%)	NA	513 (24.8%)	NA
2	1297 (42.7%)	45 (5.9%)	1391 (43%)	46 (6.1%)	1104 (44.5%)	28 (5%)	1189 (44.8%)	17 (4.4%)	935 (45.3%)	28 (5.4%)
3-5	1013 (33.3%)	681 (89.7%)	1034 (31.9%)	673 (88.7%)	754 (30.4%)	501 (89.9%)	752 (28.3%)	350 (90.7%)	591 (28.6%)	463 (89.7%)
6+	56 (1.8%)	33 (4.3%)	41 (1.3%)	40 (5.3%)	35 (1.4%)	28 (5%)	31 (1.2%)	19 (4.9%)	27 (1.3%)	25 (4.8%)
**Panel**	E	E	F	F	E	E	F	F	E	E

Note: Wave A refers to CoMix surveys collecting behavioural data for adults whereas Wave C refers to CoMix surveys collecting behavioural data from adults responding on behalf of a randomly selected child.

Table S1 (continued) CoMix sample characteristics for the United Kingdom.

Characteristic	Wave A 25	Wave C 25	Wave A 26	Wave C 26	Wave A 27	Wave C 27	Wave A 28	Wave C 28	Wave A 29	Wave C 29
**All**	2071	518	2592	675	2501	625	2216	554	2218	554
**Age**										
0-4	NA	74 (14.3%)	NA	123 (18.2%)	NA	79 (12.6%)	NA	102 (18.4%)	NA	91 (16.4%)
5-11	NA	201 (38.8%)	NA	229 (33.9%)	NA	227 (36.3%)	NA	198 (35.7%)	NA	196 (35.4%)
12-17	NA	219 (42.3%)	NA	277 (41%)	NA	285 (45.6%)	NA	206 (37.2%)	NA	241 (43.5%)
18-29	231 (11.2%)	NA	372 (14.4%)	NA	306 (12.2%)	NA	331 (14.9%)	NA	222 (10%)	NA
30-39	291 (14.1%)	NA	395 (15.2%)	NA	381 (15.2%)	NA	318 (14.4%)	NA	319 (14.4%)	NA
40-49	327 (15.8%)	NA	440 (17%)	NA	402 (16.1%)	NA	394 (17.8%)	NA	339 (15.3%)	NA
50-59	426 (20.6%)	NA	487 (18.8%)	NA	514 (20.6%)	NA	388 (17.5%)	NA	483 (21.8%)	NA
60-69	437 (21.1%)	NA	567 (21.9%)	NA	510 (20.4%)	NA	546 (24.6%)	NA	473 (21.3%)	NA
70+	359 (17.3%)	NA	331 (12.8%)	NA	388 (15.5%)	NA	239 (10.8%)	NA	382 (17.2%)	NA
Unknown age	NA	24 (4.6%)	NA	46 (6.8%)	NA	34 (5.4%)	NA	48 (8.7%)	NA	26 (4.7%)
**Gender**										
Female	1066 (51.5%)	258 (49.8%)	1371 (52.9%)	312 (46.2%)	1306 (52.2%)	331 (53%)	1152 (52%)	254 (45.8%)	1157 (52.2%)	278 (50.2%)
Male	998 (48.2%)	259 (50%)	1216 (46.9%)	359 (53.2%)	1190 (47.6%)	291 (46.6%)	1052 (47.5%)	297 (53.6%)	1058 (47.7%)	273 (49.3%)
Other	7 (0.3%)	1 (0.2%)	5 (0.2%)	4 (0.6%)	5 (0.2%)	3 (0.5%)	12 (0.5%)	3 (0.5%)	3 (0.1%)	3 (0.5%)
**Day**										
Sunday	1 (0%)	NA	118 (4.6%)	51 (7.6%)	160 (6.4%)	37 (5.9%)	121 (5.5%)	47 (8.5%)	26 (1.2%)	11 (2%)
Monday	680 (32.8%)	123 (23.7%)	476 (18.4%)	162 (24%)	406 (16.2%)	100 (16%)	288 (13%)	69 (12.5%)	1185 (53.4%)	242 (43.7%)
Tuesday	1109 (53.5%)	157 (30.3%)	111 (4.3%)	28 (4.1%)	418 (16.7%)	105 (16.8%)	173 (7.8%)	40 (7.2%)	354 (16%)	90 (16.2%)
Wednesday	241 (11.6%)	199 (38.4%)	NA	NA	NA	100 (16%)	85 (3.8%)	NA	5 (0.2%)	65 (11.7%)
Thursday	5 (0.2%)	28 (5.4%)	161 (6.2%)	32 (4.7%)	NA	9 (1.4%)	NA	NA	129 (5.8%)	53 (9.6%)
Friday	30 (1.4%)	7 (1.4%)	1510 (58.3%)	346 (51.3%)	497 (19.9%)	96 (15.4%)	1194 (53.9%)	313 (56.5%)	455 (20.5%)	79 (14.3%)
Saturday	5 (0.2%)	4 (0.8%)	216 (8.3%)	56 (8.3%)	1020 (40.8%)	178 (28.5%)	355 (16%)	85 (15.3%)	64 (2.9%)	14 (2.5%)
**Data collection**										
First day	11 Sep 20	11 Sep 20	17 Sep 20	17 Sep 20	25 Sep 20	25 Sep 20	02 Oct 20	02 Oct 20	08 Oct 20	08 Oct 20
Last day	17 Sep 20	17 Sep 20	22 Sep 20	22 Sep 20	29 Sep 20	01 Oct 20	07 Oct 20	06 Oct 20	14 Oct 20	15 Oct 20
**Household size**										
1	548 (26.5%)	NA	628 (24.2%)	NA	633 (25.3%)	NA	527 (23.8%)	NA	568 (25.6%)	NA
2	953 (46%)	31 (6%)	1146 (44.2%)	40 (5.9%)	1135 (45.4%)	43 (6.9%)	1030 (46.5%)	29 (5.2%)	1040 (46.9%)	31 (5.6%)
3-5	549 (26.5%)	460 (88.8%)	780 (30.1%)	602 (89.2%)	703 (28.1%)	553 (88.5%)	629 (28.4%)	499 (90.1%)	586 (26.4%)	497 (89.7%)
6+	21 (1%)	27 (5.2%)	38 (1.5%)	33 (4.9%)	30 (1.2%)	29 (4.6%)	30 (1.4%)	26 (4.7%)	24 (1.1%)	26 (4.7%)
**Panel**	F	F	E	E	F	F	E	E	F	F

Note: Wave A refers to CoMix surveys collecting behavioural data for adults whereas Wave C refers to CoMix surveys collecting behavioural data from adults responding on behalf of a randomly selected child.

Table S1 (continued) CoMix sample characteristics for the United Kingdom.

Characteristic	Wave A 30	Wave C 30	Wave A 31	Wave C 31	Wave A 32	Wave C 32	Wave A 33	Wave C 33	Wave A 34	Wave C 34
**All**	2389	596	2377	597	2030	502	2034	508	2292	572
**Age**										
0-4	NA	112 (18.8%)	NA	89 (14.9%)	NA	98 (19.5%)	NA	81 (15.9%)	NA	70 (12.2%)
5-11	NA	208 (34.9%)	NA	216 (36.2%)	NA	183 (36.5%)	NA	175 (34.4%)	NA	200 (35%)
12-17	NA	231 (38.8%)	NA	269 (45.1%)	NA	201 (40%)	NA	225 (44.3%)	NA	273 (47.7%)
18-29	304 (12.7%)	NA	260 (10.9%)	NA	325 (16%)	NA	184 (9%)	NA	240 (10.5%)	NA
30-39	357 (14.9%)	NA	335 (14.1%)	NA	355 (17.5%)	NA	273 (13.4%)	NA	270 (11.8%)	NA
40-49	410 (17.2%)	NA	373 (15.7%)	NA	364 (17.9%)	NA	315 (15.5%)	NA	373 (16.3%)	NA
50-59	460 (19.3%)	NA	499 (21%)	NA	361 (17.8%)	NA	433 (21.3%)	NA	461 (20.1%)	NA
60-69	553 (23.1%)	NA	510 (21.5%)	NA	374 (18.4%)	NA	451 (22.2%)	NA	570 (24.9%)	NA
70+	305 (12.8%)	NA	400 (16.8%)	NA	251 (12.4%)	NA	378 (18.6%)	NA	378 (16.5%)	NA
Unknown age	NA	45 (7.6%)	NA	23 (3.9%)	NA	20 (4%)	NA	27 (5.3%)	NA	29 (5.1%)
**Gender**										
Female	1256 (52.6%)	276 (46.3%)	1247 (52.5%)	298 (49.9%)	1078 (53.1%)	244 (48.6%)	1080 (53.1%)	259 (51%)	1146 (50%)	275 (48.1%)
Male	1129 (47.3%)	319 (53.5%)	1126 (47.4%)	296 (49.6%)	944 (46.5%)	257 (51.2%)	947 (46.6%)	247 (48.6%)	1143 (49.9%)	293 (51.2%)
Other	4 (0.2%)	1 (0.2%)	4 (0.2%)	3 (0.5%)	8 (0.4%)	1 (0.2%)	7 (0.3%)	2 (0.4%)	3 (0.1%)	4 (0.7%)
**Day**										
Sunday	131 (5.5%)	42 (7%)	706 (29.7%)	156 (26.1%)	59 (2.9%)	28 (5.6%)	44 (2.2%)	15 (3%)	308 (13.4%)	38 (6.6%)
Monday	241 (10.1%)	64 (10.7%)	193 (8.1%)	57 (9.5%)	98 (4.8%)	32 (6.4%)	281 (13.8%)	72 (14.2%)	266 (11.6%)	56 (9.8%)
Tuesday	662 (27.7%)	147 (24.7%)	828 (34.8%)	152 (25.5%)	60 (3%)	15 (3%)	NA	104 (20.5%)	52 (2.3%)	54 (9.4%)
Wednesday	35 (1.5%)	NA	417 (17.5%)	172 (28.8%)	659 (32.5%)	166 (33.1%)	NA	3 (0.6%)	NA	135 (23.6%)
Thursday	NA	NA	172 (7.2%)	43 (7.2%)	77 (3.8%)	39 (7.8%)	761 (37.4%)	147 (28.9%)	594 (25.9%)	121 (21.2%)
Friday	948 (39.7%)	247 (41.4%)	50 (2.1%)	15 (2.5%)	822 (40.5%)	171 (34.1%)	835 (41.1%)	144 (28.3%)	544 (23.7%)	111 (19.4%)
Saturday	372 (15.6%)	96 (16.1%)	11 (0.5%)	2 (0.3%)	255 (12.6%)	51 (10.2%)	113 (5.6%)	23 (4.5%)	528 (23%)	57 (10%)
**Data collection**										
First day	16 Oct 20	16 Oct 20	23 Oct 20	23 Oct 20	30 Oct 20	30 Oct 20	05 Nov 20	05 Nov 20	12 Nov 20	12 Nov 20
Last day	21 Oct 20	20 Oct 20	29 Oct 20	29 Oct 20	05 Nov 20	05 Nov 20	09 Nov 20	11 Nov 20	17 Nov 20	19 Nov 20
**Household size**										
1	587 (24.6%)	NA	623 (26.2%)	NA	476 (23.4%)	NA	554 (27.2%)	NA	583 (25.4%)	NA
2	1105 (46.3%)	29 (4.9%)	1121 (47.2%)	40 (6.7%)	870 (42.9%)	38 (7.6%)	967 (47.5%)	28 (5.5%)	1099 (47.9%)	42 (7.3%)
3-5	670 (28%)	539 (90.4%)	611 (25.7%)	530 (88.8%)	645 (31.8%)	443 (88.2%)	497 (24.4%)	455 (89.6%)	585 (25.5%)	510 (89.2%)
6+	27 (1.1%)	28 (4.7%)	22 (0.9%)	27 (4.5%)	39 (1.9%)	21 (4.2%)	16 (0.8%)	25 (4.9%)	25 (1.1%)	20 (3.5%)
**Panel**	E	E	F	F	E	E	F	F	E	E

Note: Wave A refers to CoMix surveys collecting behavioural data for adults whereas Wave C refers to CoMix surveys collecting behavioural data from adults responding on behalf of a randomly selected child.

Table S1 (continued) CoMix sample characteristics for the United Kingdom.

Characteristic	Wave A 35	Wave C 35	Wave A 36	Wave C 36	Wave A 37	Wave C 37	Wave A 38	Wave C 38	Wave A 39	Wave C 39
**All**	2290	565	2174	544	2355	592	2434	611	2357	563
**Age**										
0-4	NA	82 (14.5%)	NA	107 (19.7%)	NA	104 (17.6%)	NA	116 (19%)	NA	54 (9.6%)
5-11	NA	201 (35.6%)	NA	188 (34.6%)	NA	223 (37.7%)	NA	205 (33.6%)	NA	200 (35.5%)
12-17	NA	254 (45%)	NA	224 (41.2%)	NA	237 (40%)	NA	259 (42.4%)	NA	295 (52.4%)
18-29	225 (9.8%)	NA	284 (13.1%)	NA	368 (15.6%)	NA	341 (14%)	NA	304 (12.9%)	NA
30-39	317 (13.8%)	NA	335 (15.4%)	NA	387 (16.4%)	NA	415 (17.1%)	NA	351 (14.9%)	NA
40-49	364 (15.9%)	NA	347 (16%)	NA	391 (16.6%)	NA	412 (16.9%)	NA	392 (16.6%)	NA
50-59	490 (21.4%)	NA	418 (19.2%)	NA	456 (19.4%)	NA	478 (19.6%)	NA	494 (21%)	NA
60-69	493 (21.5%)	NA	470 (21.6%)	NA	435 (18.5%)	NA	434 (17.8%)	NA	489 (20.7%)	NA
70+	401 (17.5%)	NA	320 (14.7%)	NA	318 (13.5%)	NA	354 (14.5%)	NA	327 (13.9%)	NA
Unknown age	NA	28 (5%)	NA	25 (4.6%)	NA	28 (4.7%)	NA	31 (5.1%)	NA	14 (2.5%)
**Gender**										
Female	1205 (52.6%)	288 (51%)	1135 (52.2%)	252 (46.3%)	1204 (51.1%)	298 (50.3%)	1286 (52.8%)	290 (47.5%)	1232 (52.3%)	265 (47.1%)
Male	1081 (47.2%)	275 (48.7%)	1034 (47.6%)	289 (53.1%)	1140 (48.4%)	293 (49.5%)	1139 (46.8%)	318 (52%)	1117 (47.4%)	295 (52.4%)
Other	4 (0.2%)	2 (0.4%)	5 (0.2%)	3 (0.6%)	11 (0.5%)	1 (0.2%)	9 (0.4%)	3 (0.5%)	8 (0.3%)	3 (0.5%)
**Day**										
Sunday	66 (2.9%)	25 (4.4%)	171 (7.9%)	NA	40 (1.7%)	12 (2%)	120 (4.9%)	44 (7.2%)	NA	NA
Monday	306 (13.4%)	113 (20%)	NA	NA	208 (8.8%)	78 (13.2%)	195 (8%)	76 (12.4%)	917 (38.9%)	265 (47.1%)
Tuesday	57 (2.5%)	51 (9%)	NA	NA	590 (25.1%)	230 (38.9%)	732 (30.1%)	35 (5.7%)	1184 (50.2%)	256 (45.5%)
Wednesday	NA	NA	NA	NA	679 (28.8%)	13 (2.2%)	19 (0.8%)	3 (0.5%)	256 (10.9%)	42 (7.5%)
Thursday	150 (6.6%)	8 (1.4%)	84 (3.9%)	35 (6.4%)	28 (1.2%)	7 (1.2%)	508 (20.9%)	222 (36.3%)	NA	NA
Friday	1485 (64.8%)	315 (55.8%)	1678 (77.2%)	458 (84.2%)	728 (30.9%)	230 (38.9%)	523 (21.5%)	168 (27.5%)	NA	NA
Saturday	226 (9.9%)	53 (9.4%)	241 (11.1%)	51 (9.4%)	82 (3.5%)	22 (3.7%)	337 (13.8%)	63 (10.3%)	NA	NA
**Data collection**										
First day	19 Nov 20	19 Nov 20	26 Nov 20	26 Nov 20	03 Dec 20	03 Dec 20	10 Dec 20	10 Dec 20	21 Dec 20	21 Dec 20
Last day	24 Nov 20	24 Nov 20	29 Nov 20	28 Nov 20	09 Dec 20	09 Dec 20	16 Dec 20	16 Dec 20	23 Dec 20	23 Dec 20
**Household size**										
1	600 (26.2%)	NA	522 (24%)	NA	575 (24.4%)	NA	562 (23.1%)	NA	568 (24.1%)	NA
2	1081 (47.2%)	29 (5.1%)	997 (45.9%)	38 (7%)	1046 (44.4%)	42 (7.1%)	1064 (43.7%)	42 (6.9%)	1072 (45.5%)	40 (7.1%)
3-5	586 (25.6%)	516 (91.3%)	632 (29.1%)	489 (89.9%)	709 (30.1%)	520 (87.8%)	768 (31.6%)	547 (89.5%)	692 (29.4%)	492 (87.4%)
6+	23 (1%)	20 (3.5%)	23 (1.1%)	17 (3.1%)	25 (1.1%)	30 (5.1%)	40 (1.6%)	22 (3.6%)	25 (1.1%)	31 (5.5%)
**Panel**	F	F	E	E	F	F	E	E	F	F

Note: Wave A refers to CoMix surveys collecting behavioural data for adults whereas Wave C refers to CoMix surveys collecting behavioural data from adults responding on behalf of a randomly selected child.

Table S1 (continued) CoMix sample characteristics for the United Kingdom.

Characteristic	Wave A 40	Wave C 40	Wave A 41	Wave C 41	Wave A 42	Wave C 42	Wave A 43	Wave C 43	Wave A 44	Wave C 44
**All**	2549	638	2549	635	2014	502	2012	503	2000	501
**Age**										
0-4	NA	74 (11.6%)	NA	58 (9.1%)	NA	68 (13.5%)	NA	67 (13.3%)	NA	86 (17.2%)
5-11	NA	231 (36.2%)	NA	226 (35.6%)	NA	157 (31.3%)	NA	188 (37.4%)	NA	188 (37.5%)
12-17	NA	311 (48.7%)	NA	322 (50.7%)	NA	253 (50.4%)	NA	220 (43.7%)	NA	208 (41.5%)
18-29	334 (13.1%)	NA	339 (13.3%)	NA	227 (11.3%)	NA	425 (21.1%)	NA	252 (12.6%)	NA
30-39	400 (15.7%)	NA	420 (16.5%)	NA	341 (16.9%)	NA	416 (20.7%)	NA	360 (18%)	NA
40-49	401 (15.7%)	NA	412 (16.2%)	NA	322 (16%)	NA	324 (16.1%)	NA	328 (16.4%)	NA
50-59	535 (21%)	NA	512 (20.1%)	NA	394 (19.6%)	NA	347 (17.2%)	NA	399 (20%)	NA
60-69	516 (20.2%)	NA	531 (20.8%)	NA	421 (20.9%)	NA	301 (15%)	NA	382 (19.1%)	NA
70+	363 (14.2%)	NA	335 (13.1%)	NA	309 (15.3%)	NA	199 (9.9%)	NA	279 (14%)	NA
Unknown age	NA	22 (3.4%)	NA	29 (4.6%)	NA	24 (4.8%)	NA	28 (5.6%)	NA	19 (3.8%)
**Gender**										
Female	NA	304 (47.6%)	1371 (53.8%)	317 (49.9%)	1063 (52.8%)	248 (49.4%)	1053 (52.3%)	245 (48.7%)	1070 (53.5%)	246 (49.1%)
Male	1 (0%)	327 (51.3%)	1171 (45.9%)	314 (49.4%)	941 (46.7%)	253 (50.4%)	948 (47.1%)	254 (50.5%)	921 (46.1%)	253 (50.5%)
Other	2548 (100%)	7 (1.1%)	7 (0.3%)	4 (0.6%)	10 (0.5%)	1 (0.2%)	11 (0.5%)	4 (0.8%)	9 (0.4%)	2 (0.4%)
**Day**										
Sunday	764 (30%)	159 (24.9%)	174 (6.8%)	79 (12.4%)	NA	NA	132 (6.6%)	31 (6.2%)	100 (5%)	32 (6.4%)
Monday	177 (6.9%)	34 (5.3%)	144 (5.6%)	41 (6.5%)	NA	NA	481 (23.9%)	91 (18.1%)	507 (25.4%)	115 (23%)
Tuesday	NA	28 (4.4%)	334 (13.1%)	43 (6.8%)	NA	NA	1 (0%)	NA	212 (10.6%)	71 (14.2%)
Wednesday	NA	NA	89 (3.5%)	37 (5.8%)	481 (23.9%)	154 (30.7%)	NA	NA	5 (0.2%)	NA
Thursday	992 (38.9%)	217 (34%)	627 (24.6%)	134 (21.1%)	1282 (63.7%)	233 (46.4%)	751 (37.3%)	197 (39.2%)	NA	NA
Friday	108 (4.2%)	39 (6.1%)	814 (31.9%)	171 (26.9%)	251 (12.5%)	109 (21.7%)	452 (22.5%)	129 (25.6%)	962 (48.1%)	211 (42.1%)
Saturday	508 (19.9%)	161 (25.2%)	367 (14.4%)	130 (20.5%)	NA	6 (1.2%)	195 (9.7%)	55 (10.9%)	214 (10.7%)	72 (14.4%)
**Data collection**										
First day	24 Dec 20	24 Dec 20	30 Dec 20	30 Dec 20	06 Jan 21	06 Jan 21	14 Jan 21	14 Jan 21	22 Jan 21	22 Jan 21
Last day	28 Dec 20	29 Dec 20	06 Jan 21	06 Jan 21	08 Jan 21	09 Jan 21	19 Jan 21	18 Jan 21	27 Jan 21	26 Jan 21
**Household size**										
1	577 (22.6%)	NA	604 (23.7%)	NA	440 (21.8%)	NA	445 (22.1%)	NA	461 (23%)	NA
2	1163 (45.6%)	38 (6%)	1131 (44.4%)	45 (7.1%)	942 (46.8%)	35 (7%)	846 (42%)	42 (8.3%)	914 (45.7%)	39 (7.8%)
3-5	777 (30.5%)	583 (91.4%)	784 (30.8%)	567 (89.3%)	599 (29.7%)	452 (90%)	701 (34.8%)	437 (86.9%)	601 (30%)	451 (90%)
6+	32 (1.3%)	17 (2.7%)	30 (1.2%)	23 (3.6%)	33 (1.6%)	15 (3%)	20 (1%)	24 (4.8%)	24 (1.2%)	11 (2.2%)
**Panel**	E	E	F	F	E	E	F	F	E	E

Note: Wave A refers to CoMix surveys collecting behavioural data for adults whereas Wave C refers to CoMix surveys collecting behavioural data from adults responding on behalf of a randomly selected child.

Table S1 (continued) CoMix sample characteristics for the United Kingdom.

Characteristic	Wave A 45	Wave C 45	Wave A 46	Wave C 46	Wave A 47	Wave C 47	Wave A 48	Wave C 48	Wave A 49	Wave C 49
**All**	1994	499	1992	501	1997	500	1998	498	2000	499
**Age**										
0-4	NA	75 (15%)	NA	77 (15.4%)	NA	68 (13.6%)	NA	77 (15.5%)	NA	70 (14%)
5-11	NA	182 (36.5%)	NA	192 (38.3%)	NA	185 (37%)	NA	191 (38.4%)	NA	178 (35.7%)
12-17	NA	210 (42.1%)	NA	208 (41.5%)	NA	222 (44.4%)	NA	210 (42.2%)	NA	222 (44.5%)
18-29	384 (19.3%)	NA	247 (12.4%)	NA	364 (18.2%)	NA	232 (11.6%)	NA	313 (15.7%)	NA
30-39	372 (18.7%)	NA	345 (17.3%)	NA	375 (18.8%)	NA	347 (17.4%)	NA	361 (18%)	NA
40-49	331 (16.6%)	NA	354 (17.8%)	NA	328 (16.4%)	NA	322 (16.1%)	NA	333 (16.7%)	NA
50-59	322 (16.1%)	NA	420 (21.1%)	NA	417 (20.9%)	NA	389 (19.5%)	NA	367 (18.4%)	NA
60-69	369 (18.5%)	NA	441 (22.1%)	NA	351 (17.6%)	NA	419 (21%)	NA	384 (19.2%)	NA
70+	216 (10.8%)	NA	185 (9.3%)	NA	162 (8.1%)	NA	289 (14.5%)	NA	242 (12.1%)	NA
Unknown age	NA	32 (6.4%)	NA	24 (4.8%)	NA	25 (5%)	NA	20 (4%)	NA	29 (5.8%)
**Gender**										
Female	1047 (52.5%)	258 (51.7%)	1112 (55.8%)	249 (49.7%)	1034 (51.8%)	248 (49.6%)	1094 (54.8%)	232 (46.6%)	1034 (51.7%)	244 (48.9%)
Male	939 (47.1%)	237 (47.5%)	871 (43.7%)	250 (49.9%)	954 (47.8%)	251 (50.2%)	895 (44.8%)	262 (52.6%)	954 (47.7%)	252 (50.5%)
Other	8 (0.4%)	4 (0.8%)	9 (0.5%)	2 (0.4%)	9 (0.5%)	1 (0.2%)	9 (0.5%)	4 (0.8%)	12 (0.6%)	3 (0.6%)
**Day**										
Sunday	8 (0.4%)	15 (3%)	135 (6.8%)	37 (7.4%)	118 (5.9%)	31 (6.2%)	105 (5.3%)	37 (7.4%)	92 (4.6%)	31 (6.2%)
Monday	NA	10 (2%)	260 (13.1%)	77 (15.4%)	214 (10.7%)	51 (10.2%)	135 (6.8%)	35 (7%)	519 (26%)	86 (17.2%)
Tuesday	NA	NA	107 (5.4%)	39 (7.8%)	141 (7.1%)	14 (2.8%)	302 (15.1%)	59 (11.8%)	116 (5.8%)	47 (9.4%)
Wednesday	NA	NA	254 (12.8%)	42 (8.4%)	1 (0.1%)	NA	125 (6.3%)	23 (4.6%)	2 (0.1%)	17 (3.4%)
Thursday	1059 (53.1%)	294 (58.9%)	382 (19.2%)	112 (22.4%)	720 (36.1%)	178 (35.6%)	852 (42.6%)	203 (40.8%)	747 (37.4%)	201 (40.3%)
Friday	562 (28.2%)	118 (23.6%)	426 (21.4%)	100 (20%)	516 (25.8%)	126 (25.2%)	308 (15.4%)	79 (15.9%)	399 (20%)	94 (18.8%)
Saturday	365 (18.3%)	62 (12.4%)	428 (21.5%)	94 (18.8%)	287 (14.4%)	100 (20%)	171 (8.6%)	62 (12.4%)	125 (6.2%)	23 (4.6%)
**Data collection**										
First day	28 Jan 21	28 Jan 21	04 Feb 21	04 Feb 21	11 Feb 21	11 Feb 21	18 Feb 21	18 Feb 21	25 Feb 21	25 Feb 21
Last day	31 Jan 21	01 Feb 21	10 Feb 21	10 Feb 21	17 Feb 21	16 Feb 21	25 Feb 21	24 Feb 21	03 Mar 21	03 Mar 21
**Household size**										
1	468 (23.5%)	NA	451 (22.6%)	NA	484 (24.2%)	NA	453 (22.7%)	1 (0.2%)	507 (25.4%)	NA
2	851 (42.7%)	38 (7.6%)	883 (44.3%)	45 (9%)	842 (42.2%)	39 (7.8%)	893 (44.7%)	38 (7.6%)	851 (42.5%)	36 (7.2%)
3-5	656 (32.9%)	432 (86.6%)	619 (31.1%)	438 (87.4%)	648 (32.4%)	432 (86.4%)	621 (31.1%)	440 (88.4%)	624 (31.2%)	437 (87.6%)
6+	19 (1%)	29 (5.8%)	39 (2%)	18 (3.6%)	23 (1.2%)	29 (5.8%)	31 (1.6%)	19 (3.8%)	18 (0.9%)	26 (5.2%)
**Panel**	F	F	E	E	F	F	E	E	F	F

Note: Wave A refers to CoMix surveys collecting behavioural data for adults whereas Wave C refers to CoMix surveys collecting behavioural data from adults responding on behalf of a randomly selected child.

Table S1 (continued) CoMix sample characteristics for the United Kingdom.

Characteristic	Wave A 50	Wave C 50	Wave A 51	Wave C 51	Wave A 52	Wave C 52	Wave A 53	Wave C 53
**All**	1996	500	1999	502	1997	502	1994	489
**Age**								
0-4	NA	87 (17.4%)	NA	74 (14.7%)	NA	73 (14.5%)	NA	84 (17.2%)
5-11	NA	193 (38.6%)	NA	188 (37.5%)	NA	196 (39%)	NA	168 (34.4%)
12-17	NA	204 (40.8%)	NA	214 (42.6%)	NA	211 (42%)	NA	209 (42.7%)
18-29	203 (10.2%)	NA	289 (14.5%)	NA	201 (10.1%)	NA	314 (15.7%)	NA
30-39	303 (15.2%)	NA	372 (18.6%)	NA	306 (15.3%)	NA	358 (18%)	NA
40-49	322 (16.1%)	NA	302 (15.1%)	NA	320 (16%)	NA	301 (15.1%)	NA
50-59	397 (19.9%)	NA	377 (18.9%)	NA	428 (21.4%)	NA	388 (19.5%)	NA
60-69	454 (22.7%)	NA	396 (19.8%)	NA	448 (22.4%)	NA	385 (19.3%)	NA
70+	317 (15.9%)	NA	263 (13.2%)	NA	294 (14.7%)	NA	248 (12.4%)	NA
Unknown age	NA	16 (3.2%)	NA	26 (5.2%)	NA	22 (4.4%)	NA	28 (5.7%)
**Gender**								
Female	1091 (54.7%)	244 (48.8%)	1024 (51.2%)	244 (48.6%)	1038 (52%)	248 (49.4%)	1020 (51.2%)	248 (50.7%)
Male	897 (44.9%)	253 (50.6%)	964 (48.2%)	256 (51%)	953 (47.7%)	252 (50.2%)	960 (48.1%)	239 (48.9%)
Other	8 (0.4%)	3 (0.6%)	11 (0.6%)	2 (0.4%)	6 (0.3%)	2 (0.4%)	14 (0.7%)	2 (0.4%)
**Day**								
Sunday	107 (5.4%)	33 (6.6%)	122 (6.1%)	55 (11%)	77 (3.9%)	25 (5%)	187 (9.4%)	48 (9.8%)
Monday	284 (14.2%)	41 (8.2%)	101 (5.1%)	47 (9.4%)	260 (13%)	49 (9.8%)	241 (12.1%)	58 (11.9%)
Tuesday	135 (6.8%)	23 (4.6%)	298 (14.9%)	47 (9.4%)	346 (17.3%)	53 (10.6%)	683 (34.3%)	189 (38.7%)
Wednesday	65 (3.3%)	52 (10.4%)	24 (1.2%)	156 (31.1%)	144 (7.2%)	120 (23.9%)	NA	NA
Thursday	798 (40%)	206 (41.2%)	NA	NA	580 (29%)	127 (25.3%)	50 (2.5%)	36 (7.4%)
Friday	414 (20.7%)	86 (17.2%)	1152 (57.6%)	103 (20.5%)	404 (20.2%)	87 (17.3%)	534 (26.8%)	111 (22.7%)
Saturday	193 (9.7%)	59 (11.8%)	302 (15.1%)	94 (18.7%)	186 (9.3%)	41 (8.2%)	299 (15%)	47 (9.6%)
**Data collection**								
First day	04 Mar 21	04 Mar 21	12 Mar 21	12 Mar 21	18 Mar 21	18 Mar 21	25 Mar 21	25 Mar 21
Last day	11 Mar 21	11 Mar 21	17 Mar 21	17 Mar 21	24 Mar 21	24 Mar 21	30 Mar 21	30 Mar 21
**Household size**								
1	456 (22.8%)	NA	499 (25%)	NA	442 (22.1%)	NA	469 (23.5%)	NA
2	913 (45.7%)	42 (8.4%)	889 (44.5%)	42 (8.4%)	918 (46%)	36 (7.2%)	879 (44.1%)	33 (6.7%)
3-5	613 (30.7%)	442 (88.4%)	588 (29.4%)	429 (85.5%)	610 (30.5%)	448 (89.2%)	623 (31.2%)	437 (89.4%)
6+	14 (0.7%)	16 (3.2%)	23 (1.2%)	31 (6.2%)	27 (1.4%)	18 (3.6%)	23 (1.2%)	19 (3.9%)
**Panel**	E	E	F	F	E	E	F	F

Note: Wave A refers to CoMix surveys collecting behavioural data for adults whereas Wave C refers to CoMix surveys collecting behavioural data from adults responding on behalf of a randomly selected child.

1.2 Belgium

Table S2. CoMix sample characteristics for Belgium, Waves 9 to 25

Characteristic	Wave 9	Wave 10	Wave 11	Wave 12	Wave 13	Wave 14	Wave 15	Wave 16	Wave 17	Wave 18
**All**	1500	1340	1209	1087	983	883	799	719	645	1497
**Age**										
0-4	48 (3.3%)	41 (3.1%)	35 (2.9%)	34 (3.1%)	33 (3.4%)	35 (4.0%)	28 (3.5%)	22 (3.1%)	18 (2.8%)	51 (3.4%)
5-11	115 (7.7%)	91 (6.8%)	88 (7.3%)	79 (7.3%)	72 (7.3%)	67 (7.6%)	51 (6.4%)	50 (7.0%)	54 (8.4%)	108 (7.2%)
12-15	87 (5.8%)	77 (5.7%)	73 (6.0%)	70 (6.4%)	55 (5.6%)	45 (5.1%)	49 (6.1%)	44 (6.1%)	31 (4.8%)	83 (5.5%)
16-17	50 (3.3%)	50 (3.7%)	40 (3.3%)	34 (3.1%)	34 (3.5%)	26 (2.9%)	28 (3.5%)	25 (3.5%)	19 (2.9%)	50 (3.3%)
18-29	228 (15.2%)	180 (13.4%)	137 (11.3%)	126 (11.6%)	137 (13.9%)	137 (15.5%)	100 (12.5%)	84 (11.7%)	83 (12.9%)	221 (14.8%)
30-39	174 (11.6%)	125 (9.3%)	125 (10.3%)	116 (10.7%)	113 (11.5%)	121 (13.7%)	81 (10.1%)	73 (10.2%)	76 (11.8%)	188 (12.6%)
40-49	195 (13.0%)	156 (11.6%)	173 (14.3%)	155 (14.3%)	136 (13.8%)	156 (17.7%)	114 (14.3%)	100 (13.9%)	87 (13.5%)	191 (12.8%)
50-59	191 (12.7%)	215 (16.0%)	176 (14.6%)	160 (14.7%)	137 (13.9%)	118 (13.4%)	121 (15.1%)	138 (19.2%)	89 (13.8%)	201 (13.4%)
60-69	285 (19.0%)	276 (20.6%)	249 (20.6%)	220 (20.2%)	184 (18.7%)	133 (15.1%)	145 (18.1%)	135 (18.8%)	156 (24.2%)	267 (17.8%)
70-120	127 (8.5%)	125 (9.3%)	107 (8.9%)	90 (8.3%)	79 (8.0%)	40 (4.5%)	78 (9.8%)	45 (6.3%)	24 (3.7%)	130 (8.7%)
NA/Other	NA	4 (0.3%)	6 (0.5%)	3 (0.3%)	3 (0.3%)	5 (0.6%)	4 (0.5%)	3 (0.4%)	8 (1.2%)	7 (0.5%)
**Gender**										
Female	740 (49.3%)	663 (49.5%)	582 (48.1%)	520 (47.8%)	487 (49.5%)	449 (50.8%)	399 (49.9%)	346 (48.1%)	317 (49.1%)	729 (48.7%)
Male	759 (50.6%)	675 (50.4%)	627 (51.9%)	566 (52.1%)	496 (50.5%)	433 (49.0%)	400 (50.1%)	372 (51.7%)	328 (50.9%)	766 (51.2%)
Other or Missing	1 (0.1%)	2 (0.1%)	NA	1 (0.1%)	NA	1 (0.1%)	NA	1 (0.1%)	NA	2 (0.1%)
0	NA	NA	NA	NA	NA	NA	NA	NA	NA	NA
**Day**										
Sunday	33 (2.2%)	315 (23.5%)	118 (9.8%)	28 (2.6%)	48 (4.9%)	1 (0.1%)	7 (0.9%)	129 (17.9%)	NA	56 (3.7%)
Monday	64 (4.3%)	71 (5.3%)	67 (5.5%)	93 (8.6%)	22 (2.2%)	NA	NA	175 (24.3%)	NA	41 (2.7%)
Tuesday	101 (6.7%)	55 (4.1%)	138 (11.4%)	107 (9.8%)	160 (16.3%)	386 (43.7%)	30 (3.8%)	39 (5.4%)	458 (71.0%)	639 (42.7%)
Wednesday	513 (34.2%)	29 (2.2%)	62 (5.1%)	675 (62.1%)	687 (69.9%)	256 (29.0%)	8 (1.0%)	62 (8.6%)	149 (23.1%)	254 (17.0%)
Thursday	666 (44.4%)	640 (47.8%)	747 (61.8%)	82 (7.5%)	34 (3.5%)	213 (24.1%)	298 (37.3%)	143 (19.9%)	38 (5.9%)	191 (12.8%)
Friday	100 (6.7%)	111 (8.3%)	59 (4.9%)	82 (7.5%)	21 (2.1%)	27 (3.1%)	380 (47.6%)	78 (10.8%)	NA	179 (12.0%)
Saturday	23 (1.5%)	119 (8.9%)	18 (1.5%)	20 (1.8%)	11 (1.1%)	NA	76 (9.5%)	93 (12.9%)	NA	137 (9.2%)
**Data collection**										
First day	12 Nov 20	27 Nov 20	10 Dec 20	23 Dec 20	06 Jan 21	20 Jan 21	03 Feb 21	17 Feb 21	03 Mar 21	17 Mar 21
Last day	19 Nov 20	09 Dec 20	17 Dec 20	05 Jan 21	12 Jan 21	25 Jan 21	08 Feb 21	24 Feb 21	05 Mar 21	25 Mar 21
**Household size**										
1	329 (21.9%)	285 (21.3%)	277 (22.9%)	251 (23.1%)	235 (23.9%)	201 (22.8%)	198 (24.8%)	167 (23.2%)	161 (25.0%)	342 (22.8%)
2	535 (35.7%)	523 (39.0%)	454 (37.6%)	419 (38.5%)	359 (36.5%)	299 (33.9%)	306 (38.3%)	273 (38.0%)	228 (35.3%)	559 (37.3%)
3	284 (18.9%)	243 (18.1%)	226 (18.7%)	195 (17.9%)	179 (18.2%)	166 (18.8%)	135 (16.9%)	127 (17.7%)	128 (19.8%)	288 (19.2%)
4	254 (16.9%)	201 (15.0%)	184 (15.2%)	160 (14.7%)	143 (14.5%)	150 (17.0%)	119 (14.9%)	109 (15.2%)	88 (13.6%)	210 (14.0%)
5+	98 (6.5%)	88 (6.6%)	68 (5.6%)	62 (5.7%)	67 (6.8%)	67 (7.6%)	41 (5.1%)	43 (6.0%)	40 (6.2%)	98 (6.5%)

Table S2 (continued) CoMix sample characteristics for Belgium, Waves 9 to 25

Characteristic	Wave 19	Wave 20	Wave 21	Wave 22	Wave 23	Wave 24	Wave 25
**All**	1383	1213	1213	1498	1201	1336	1185
**Age**							
0-4	41 (3.0%)	45 (3.7%)	36 (3.0%)	49 (3.3%)	27 (2.2%)	33 (2.5%)	30 (2.5%)
5-11	106 (7.7%)	82 (6.8%)	96 (7.9%)	110 (7.3%)	93 (7.7%)	106 (7.9%)	90 (7.6%)
12-15	81 (5.9%)	70 (5.8%)	71 (5.9%)	83 (5.5%)	78 (6.5%)	82 (6.1%)	72 (6.1%)
16-17	46 (3.3%)	39 (3.2%)	33 (2.7%)	48 (3.2%)	35 (2.9%)	41 (3.1%)	39 (3.3%)
18-29	187 (13.5%)	165 (13.6%)	158 (13.0%)	208 (13.9%)	125 (10.4%)	140 (10.5%)	123 (10.4%)
30-39	150 (10.8%)	150 (12.4%)	146 (12.0%)	169 (11.3%)	127 (10.6%)	126 (9.4%)	128 (10.8%)
40-49	175 (12.7%)	159 (13.1%)	164 (13.5%)	198 (13.2%)	158 (13.2%)	181 (13.5%)	171 (14.4%)
50-59	201 (14.5%)	183 (15.1%)	180 (14.8%)	230 (15.4%)	176 (14.7%)	203 (15.2%)	172 (14.5%)
60-69	258 (18.7%)	219 (18.1%)	211 (17.4%)	258 (17.2%)	249 (20.7%)	268 (20.1%)	234 (19.7%)
70-120	133 (9.6%)	95 (7.8%)	111 (9.2%)	136 (9.1%)	125 (10.4%)	151 (11.3%)	119 (10.0%)
NA/Other	5 (0.4%)	6 (0.5%)	7 (0.6%)	9 (0.6%)	8 (0.7%)	5 (0.4%)	7 (0.6%)
**Gender**							
Female	676 (48.9%)	592 (48.8%)	584 (48.1%)	728 (48.6%)	562 (46.8%)	664 (49.7%)	570 (48.1%)
Male	705 (51.0%)	619 (51.0%)	627 (51.7%)	767 (51.2%)	637 (53.0%)	672 (50.3%)	614 (51.8%)
Other or Missing	1 (0.1%)	2 (0.2%)	2 (0.2%)	2 (0.1%)	1 (0.1%)	NA	1 (0.1%)
0	1 (0.1%)	NA	NA	1 (0.1%)	1 (0.1%)	NA	NA
**Day**							
Sunday	216 (15.6%)	268 (22.1%)	19 (1.6%)	301 (20.1%)	370 (30.8%)	342 (25.6%)	30 (2.5%)
Monday	243 (17.6%)	225 (18.5%)	143 (11.8%)	147 (9.8%)	30 (2.5%)	40 (3.0%)	NA
Tuesday	190 (13.7%)	291 (24.0%)	647 (53.3%)	NA	40 (3.3%)	30 (2.2%)	353 (29.8%)
Wednesday	130 (9.4%)	8 (0.7%)	173 (14.3%)	215 (14.4%)	270 (22.5%)	222 (16.6%)	116 (9.8%)
Thursday	282 (20.4%)	191 (15.7%)	154 (12.7%)	360 (24.0%)	171 (14.2%)	396 (29.6%)	633 (53.4%)
Friday	251 (18.1%)	163 (13.4%)	44 (3.6%)	250 (16.7%)	187 (15.6%)	234 (17.5%)	53 (4.5%)
Saturday	71 (5.1%)	67 (5.5%)	33 (2.7%)	225 (15.0%)	133 (11.1%)	72 (5.4%)	NA
**Data collection**							
First day	31 Mar 21	14 Apr 21	28 Apr 21	13 May 21	26 May 21	10 Jun 21	23 Jun 21
Last day	07 Apr 21	20 Apr 21	04 May 21	24 May 21	02 Jun 21	17 Jun 21	28 Jun 21
**Household size**							
1	331 (23.9%)	289 (23.8%)	293 (24.2%)	341 (22.8%)	296 (24.6%)	313 (23.4%)	302 (25.5%)
2	531 (38.4%)	455 (37.5%)	449 (37.0%)	564 (37.7%)	460 (38.3%)	511 (38.2%)	441 (37.2%)
3	234 (16.9%)	224 (18.5%)	217 (17.9%)	277 (18.5%)	204 (17.0%)	233 (17.4%)	210 (17.7%)
4	201 (14.5%)	167 (13.8%)	179 (14.8%)	221 (14.8%)	170 (14.2%)	198 (14.8%)	172 (14.5%)
5+	86 (6.2%)	78 (6.4%)	75 (6.2%)	95 (6.3%)	71 (5.9%)	81 (6.1%)	60 (5.1%)

1.3 The Netherlands

Table S3. CoMix sample characteristics for the Netherlands

Characteristic	Wave A 1	Wave A 2	Wave A 3	Wave A 4	Wave A 5	Wave A 6	Wave A 7	Wave A 8	Wave A 9	Wave C 9
**All**	1560	1346	1175	1052	1157	1144	985	995	1185	298
**Age**										
0-4	NA	NA	NA	NA	NA	NA	NA	NA	NA	49 (16.4%)
5-11	NA	NA	NA	NA	NA	NA	NA	NA	NA	99 (33.2%)
12-15	NA	NA	NA	NA	NA	NA	NA	NA	NA	99 (33.2%)
16-17	NA	NA	NA	NA	NA	NA	NA	NA	NA	51 (17.1%)
18-29	194 (12.4%)	124 (9.2%)	97 (8.3%)	93 (8.8%)	91 (7.9%)	80 (7.0%)	67 (6.8%)	62 (6.2%)	341 (28.8%)	NA
30-39	229 (14.7%)	185 (13.7%)	149 (12.7%)	133 (12.6%)	142 (12.3%)	150 (13.1%)	117 (11.0%)	119 (12.0%)	181 (15.3%)	NA
40-49	267 (17.1%)	246 (18.3%)	207 (17.6%)	197 (18.7%)	213 (18.4%)	209 (18.3%)	174 (17.7%)	184 (18.5%)	160 (13.5%)	NA
50-59	269 (17.2%)	253 (18.8%)	230 (19.6%)	200 (19.0%)	231 (20.0%)	213 (18.6%)	197 (20.0%)	189 (19.0%)	185 (15.6%)	NA
60-69	408 (26.2%)	359 (26.7%)	335 (28.5%)	302 (28.7%)	329 (28.4%)	333 (29.1%)	291 (29.5%)	301 (30.3%)	219 (18.5%)	NA
70-120	193 (12.4%)	179 (13.3%)	157 (13.4%)	127 (12.1%)	151 (13.1%)	159 (13.9%)	139 (14.1%)	140 (14.1%)	99 (8.4%)	NA
**Gender**										
Female	762 (48.9%)	651 (48.4%)	568 (48.3%)	510 (48.5%)	567 (49.0%)	554 (48.4%)	490 (49.8%)	489 (49.2%)	603 (50.9%)	138 (46.3%)
Male	798 (51.2%)	695 (51.6%)	607 (51.7%)	542 (51.5%)	590 (51.0%)	590 (51.6%)	495 (50.3%)	506 (50.9%)	582 (49.1%)	160 (53.7%)
**Day**										
Sunday	640 (41.0%)	711 (52.8%)	467 (39.7%)	39 (3.7%)	113 (9.8%)	107 (9.4%)	263 (26.7%)	74 (7.4%)	13 (1.1%)	2 (0.7%)
Monday	299 (19.2%)	169 (12.6%)	142 (12.1%)	17 (1.6%)	65 (5.6%)	64 (5.6%)	57 (5.8%)	86 (8.6%)	36 (3.0%)	55 (18.5%)
Tuesday	125 (8.0%)	91 (6.8%)	69 (5.9%)	13 (1.2%)	35 (3.0%)	23 (2.0%)	108 (11.0%)	56 (5.6%)	15 (1.3%)	NA
Wednesday	20 (1.3%)	18 (1.3%)	15 (1.3%)	233 (22.2%)	240 (20.7%)	250 (21.9%)	163 (16.6%)	226 (22.7%)	213 (18.0%)	47 (15.8%)
Thursday	310 (19.9%)	242 (18.0%)	337 (28.7%)	605 (57.5%)	594 (51.3%)	555 (48.5%)	20 (2.0%)	185 (18.6%)	831 (70.1%)	182 (61.1%)
Friday	119 (7.6%)	78 (5.8%)	103 (8.8%)	105 (10.0%)	75 (6.5%)	104 (9.1%)	150 (15.2%)	167 (16.8%)	64 (5.4%)	5 (1.7%)
Saturday	47 (3.0%)	37 (2.8%)	42 (3.6%)	40 (3.8%)	35 (3.0%)	41(3.6%)	224 (22.7%)	201 (20.2%)	13 (1.1%)	7 (2.4%)
**Data collection**										
First day	16 Apr 20	07 May 20	21 May 20	03 Jun 20	17 Jun 20	01 Jul 20	15 Jul 20	29 Jul 20	23 Dec 20	23 Dec 20
Last day	26 Apr 20	14 May 20	28 May 20	10 Jun 20	24 Jun 20	08 Jul 20	22 Jul 20	05 Aug 20	03 Jan 21	28 Dec 20
**Household size**										
1	320 (20.5%)	330 (24.5%)	305 (26.0%)	282 (26.8%)	317 (27.4%)	316 (27.6%)	303 (30.8%)	315 (31.7%)	401 (33.8%)	NA
2	583 (37.4%)	507 (37.7%)	474 (40.3%)	435 (41.4%)	476 (41.1%)	504 (44.1%)	443 (45.0%)	437 (43.9%)	446 (37.6%)	20 (6.7%)
3-5	644 (41.3%)	498 (37.0%)	388 (33.0%)	330 (31.4%)	358 (30.9%)	321 (28.0%)	236 (24.0%)	241 (24.2%)	326 (27.5%)	277 (93.0%)
6+	13 (0.8%)	11 (0.8%)	8 (0.7%)	5 (0.5%)	6 (0.5%)	3 (0.3%)	3 (0.3%)	2 (0.2%)	12 (1.0%)	1 (0.3%)

Note: Wave A refers to CoMix surveys collecting behavioural data for adults whereas Wave C refers to CoMix surveys collecting behavioural data from adults responding on behalf of a randomly selected child.

Table S3 (continued) CoMix sample characteristics for the Netherlands

Characteristic	Wave A 10	Wave C 10	Wave A 11	Wave C 11	Wave A 12	Wave C 12	Wave A 13	Wave C 13	Wave A 14	Wave C 14
**All**	1076	259	968	236	873	212	783	187	703	171
**Age**										
0-4	NA	40 (15.4%)	NA	40 (17.0%)	NA	35 (16.5%)	NA	29 (15.5%)	NA	28 (16.4%)
5-11	NA	92 (35.5%)	NA	74 (31.4%)	NA	71 (33.5%)	NA	58 (31.0%)	NA	65 (38.0%)
12-15	NA	84 (32.4%)	NA	83 (35.2%)	NA	70 (33.0%)	NA	66 (35.3%)	NA	53 (31.0%)
16-17	NA	43 (16.6%)	NA	39 (16.5%)	NA	36 (17.0%)	NA	34 (18.2%)	NA	25 (14.6%)
18-29	225 (20.9%)	NA	251 (25.9%)	NA	180 (20.6%)	NA	147 (18.8%)	NA	178 (25.3%)	NA
30-39	153 (14.2%)	NA	168 (17.4%)	NA	135 (15.5%)	NA	109 (13.9%)	NA	145 (20.6%)	NA
40-49	146 (13.5%)	NA	126 (13.0%)	NA	111 (12.7%)	NA	112 (14.3%)	NA	117 (16.6%)	NA
50-59	195 (18.1%)	NA	162 (16.7%)	NA	154 (17.6%)	NA	141 (18.0%)	NA	125 (17.8%)	NA
60-69	235 (21.8%)	NA	165 (17.1%)	NA	193 (22.1%)	NA	185 (23.6%)	NA	107 (15.2%)	NA
70-120	122 (11.3%)	NA	96 (9.9%)	NA	100 (11.5%)	NA	89 (11.4%)	NA	31 (4.4%)	NA
**Gender**										
Female	528 (49.1%)	124 (47.9%)	486 (50.2%)	119 (50.4%)	421 (48.2%)	104 (49.1%)	371 (47.4%)	88 (47.1%)	353 (50.2%)	79 (46.2%)
Male	548 (50.9%)	135 (52.1%)	482 (49.8%)	117 (49.6%)	452 (51.8%)	108 (50.9%)	412 (52.6%)	99 (52.9%)	350 (49.8%)	92 (53.8%)
**Day**										
Sunday	370 (34.4%)	133 (51.4%)	689 (71.2%)	169 (71.6%)	611 (70.0%)	107 (50.5%)	133 (17.0%)	3 (1.6%)	112 (15.9%)	NA
Monday	87 (8.1%)	40 (15.4%)	123 (12.7%)	15 (6.4%)	108 (12.4%)	60 (28.3%)	408 (52.1%)	111 (59.4%)	7 (1.0%)	1 (0.6%)
Tuesday	221 (20.5%)	86 (33.2%)	15 (1.6%)	4 (1.7%)	NA	15 (7.1%)	71 (9.1%)	16 (8.6%)	NA	3 (1.8%)
Wednesday	NA	NA	133 (13.7%)	48 (20.3%)	154 (17.6%)	30 (14.2%)	15 (1.9%)	4 (2.1%)	NA	1 (0.6%)
Thursday	222 (20.6%)	NA	8 (0.8%)	NA	NA	NA	62 (7.9%)	43 (23.0%)	445 (63.3%)	147 (86.0%)
Friday	138 (12.8%)	NA	NA	NA	NA	NA	81 (10.3%)	8 (4.3%)	92 (13.1%)	19 (11.1%)
Saturday	38 (3.5%)	NA	NA	NA	NA	NA	13 (1.7%)	2 (1.1%)	47 (6.7%)	NA
**Data collection**										
First day	07 Jan 21	10 Jan 21	24 Jan 21	24 Jan 21	03 Feb 21	03 Feb 21	17 Feb 21	17 Feb 21	04 Mar 21	04 Mar 21
Last day	12 Jan 21	12 Jan 21	28 Jan 2	27 Jan 21	08 Feb 21	10 Feb 21	24 Feb 21	23 Feb 21	08 Mar 21	10 Mar 21
**Household size**										
1	362 (33.6%)	NA	334 (34.5%)	NA	299 (34.3%)	NA	273 (34.9%)	NA	267 (38.0%)	NA
2	449 (41.7%)	16 (6.2%)	377 (39.0%)	21 (8.9%)	372 (42.6%)	21 (9.9%)	341 (43.6%)	16 (8.6%)	227 (32.3%)	10 (5.9%)
3-5	262 (24.4%)	241 (93.1%)	251 (25.9%)	212 (89.8%)	201 (23.0%)	189 (89.2%)	167 (21.3%)	171 (91.4%)	208 (29.6%)	160 (93.6%)
6+	3 (0.3%)	2 (0.8%)	6 (0.6%)	3 (1.3%)	1 (0.1%)	2 (0.9%)	2 (0.3%)	NA	1 (0.1%)	1 (0.6%)

Note: Wave A refers to CoMix surveys collecting behavioural data for adults whereas Wave C refers to CoMix surveys collecting behavioural data from adults responding on behalf of a randomly selected child.

Table S3 (continued) CoMix sample characteristics for the Netherlands

Characteristic	Wave A 15	Wave C 15	Wave A 16	Wave C 16	Wave A 17	Wave C 17	Wave A 18	Wave C 18	Wave A 19	Wave C 19
**All**	636	148	631	152	509	123	461	113	1200	286
**Age**										
0-4	NA	27 (18.2%)	NA	23 (15.1%)	NA	13 (10.6%)	NA	16 (14.2%)	NA	36 (12.6%)
5-11	NA	53 (35.8%)	NA	50 (32.9%)	NA	40 (32.5%)	NA	30 (26.6%)	NA	91 (31.8%)
12-15	NA	48 (32.4%)	NA	52 (34.2%)	NA	50 (40.7%)	NA	42 (37.2%)	NA	111 (38.8%)
16-17	NA	20 (13.5%)	NA	27 (17.8%)	NA	20 (16.3%)	NA	25 (22.1%)	NA	48 (16.8%)
18-29	114 (17.9%)	NA	141 (22.4%)	NA	132 (25.9%)	NA	107 (23.2%)	NA	298 (24.8%)	NA
30-39	111 (17.5%)	NA	120 (19.0%)	NA	105 (20.6%)	NA	103 (22.3%)	NA	176 (14.7%)	NA
40-49	88 (13.8%)	NA	114 (18.1%)	NA	107 (21.0%)	NA	86 (18.7%)	NA	167 (13.9%)	NA
50-59	112 (17.6%)	NA	110 (17.4%)	NA	70 (13.8%)	NA	107 (23.2%)	NA	208 (17.3%)	NA
60-69	142 (22.3%)	NA	89 (14.1%)	NA	67 (13.2%)	NA	43 (9.3%)	NA	222 (18.5%)	NA
70-120	69 (10.9%)	NA	57 (9.0%)	NA	28 (5.5%)	NA	15 (3.3%)	NA	129 (10.8%)	NA
**Gender**										
Female	323 (50.8%)	77 (52.0%)	307 (48.7%)	75 (49.3%)	255 (50.1%)	61 (49.6%)	237 (51.4%)	51 (45.1%)	655 (54.6%)	137 (47.9%)
Male	313 (49.2%)	71 (48.0%)	324 (51.4%)	77 (50.7%)	254 (49.9%)	62 (50.4%)	224 (48.6%)	62 (54.8%)	545 (45.4%)	149 (52.1%)
**Day**										
Sunday	153 (24.1%)	22 (14.9%)	152 (24.1%)	35 (23.0%)	35 (6.9%)	7 (5.7%)	NA	NA	438 (36.5%)	56 (19.6%)
Monday	454 (71.4%)	124 (83.8%)	13 (2.1%)	NA	145 (28.5%)	NA	3 (0.7%)	NA	82 (6.8%)	27 (9.4%)
Tuesday	NA	NA	58 (9.2%)	NA	11 (2.2%)	NA	NA	NA	159 (13.3%)	52 (18.2%)
Wednesday	3 (0.5%)	NA	195 (30.9%)	36 (23.7%)	NA	NA	227 (49.2%)	64 (56.6%)	71 (5.9%)	4 (1.4%)
Thursday	10 (1.6%)	NA	129 (20.4%)	32 (21.1%)	174 (34.2%)	70 (56.9%)	192 (41.7%)	49 (43.4%)	277 (23.1%)	41 (14.3%)
Friday	1 (0.2%)	NA	51 (8.1%)	29 (19.1%)	85 (16.7%)	34 (27.6%)	38 (8.2%)	NA	92 (7.7%)	53 (18.5%)
Saturday	15 (2.4%)	2 (1.4%)	33 (5.2%)	20 (13.2%)	59 (11.6%)	12 (9.8%)	1 (0.2%)	NA	81 (6.8%)	53 (18.5%)
**Data collection**										
First day	18 Mar 21	20 Mar 21	31 Mar 21	31 Mar 21	15 Apr 21	15 Apr 21	28 Apr 21	28 Apr 21	12 May 21	12 May 21
Last day	24 Mar 21	22 Mar 21	07 Apr 21	04 Apr 21	20 Apr 21	18 Apr 21	03 May 21	29 Apr 21	23 May 21	18 May 21
**Household size**										
1	223 (35.1%)	NA	250 (39.6%)	NA	197 (38.7%)	NA	175 (38.0%)	NA	381 (31.8%)	NA
2	269 (42.3%)	11 (7.4%)	219 (34.7%)	11 (7.2%)	166 (32.6%)	10 (8.1%)	151 (32.8%)	8 (7.1%)	472 (39.3%)	17 (5.9%)
3-5	142 (22.3%)	136 (91.9%)	161 (25.5%)	140 (92.1%)	144 (28.3%)	112 (91.1%)	135 (29.3%)	104 (92.0%)	341 (28.4%)	268 (93.7%)
6+	2 (0.3%)	1 (0.7%)	1 (0.2%)	1 (0.7%)	2 (0.4%)	1 (0.8%)	NA	1 (0.9%)	6 (0.5%)	1 (0.4%)

Note: Wave A refers to CoMix surveys collecting behavioural data for adults whereas Wave C refers to CoMix surveys collecting behavioural data from adults responding on behalf of a randomly selected child.

Table S3 (continued) CoMix sample characteristics for the Netherlands

Characteristic	Wave A 20	Wave C 20	Wave A 21	Wave C 21	Wave A 22	Wave C 22	Wave A 23	Wave C 23
**All**	1054	256	961	222	861	195	771	176
**Age**								
0-4	NA	33 (12.9%)	NA	31 (14.0%)	NA	23 (11.8%)	NA	24 (13.6%)
5-11	NA	85 (33.2%)	NA	72 (32.4%)	NA	72 (36.9%)	NA	61 (34.7%)
12-15	NA	89 (34.8%)	NA	87 (39.2%)	NA	71 (36.4%)	NA	60 (34.1%)
16-17	NA	49 (19.1%)	NA	32 (14.4%)	NA	29 (14.9%)	NA	31 (17.6%)
18-29	266 (25.2%)	NA	196 (20.4%)	NA	147 (17.1%)	NA	147 (19.1%)	NA
30-39	175 (16.6%)	NA	151 (15.7%)	NA	136 (15.8%)	NA	123 (16.0%)	NA
40-49	154 (14.6%)	NA	127 (13.2%)	NA	120 (13.9%)	NA	123 (16.0%)	NA
50-59	179 (17.0%)	NA	174 (18.1%)	NA	155 (18.00%)	NA	120 (15.6%)	NA
60-69	170 (16.1%)	NA	199 (20.7%)	NA	186 (21.6%)	NA	165 (21.4%)	NA
70-120	110 (10.4%)	NA	114 (11.9%)	NA	117 (13.6%)	NA	93 (12.1%)	NA
**Gender**								
Female	580 (55.0%)	119 (46.5%)	521 (54.2%)	104 (46.9%)	438 (50.9%)	95 (48.7%)	390 (50.6%)	81 (46.0%)
Male	474 (45.0%)	137 (53.5%)	440 (45.8%)	118 (53.2%)	423 (49.1%)	100 (51.3%)	381 (49.4%)	95 (54.0%)
**Day**								
Sunday	81 (7.7%)	52 (20.3%)	269 (28.0%)	135 (60.8%)	384 (44.6%)	13 (6.7%)	133 (17.3%)	48 (27.3%)
Monday	66 (6.3%)	16 (6.3%)	64 (6.7%)	20 (9.0%)	264 (30.7%)	108 (55.4%)	290 (37.6%)	42 (23.9%)
Tuesday	57 (5.4%)	13 (5.1%)	43 (4.5%)	12 (5.4%)	68 (7.9%)	52 (26.7%)	NA	18 (10.2%)
Wednesday	79 (7.5%)	36 (14.1%)	77 (8.0%)	14 (6.3%)	46 (5.3%)	5 (2.6%)	30 (3.9%)	17 (9.7%)
Thursday	191 (18.1%)	31 (12.1%)	180 (18.7%)	27 (12.2%)	75 (8.7%)	10 (5.1%)	52 (6.7%)	23 (13.1%)
Friday	467 (44.3%)	78 (30.5%)	152 (15.8%)	5 (2.2%)	14 (1.6%)	7 (3.6%)	95 (12.3%)	9 (5.1%)
Saturday	113 (10.7%)	30 (11.7%)	176 (18.3%)	9 (4.1%)	10 (1.2%)	NA	171 (22.2%)	19 (10.8%)
**Data collection**								
First day	26 May 21	26 May 21	09 Jun 21	09 Jun 21	23 Jun 21	23 Jun 21	07 Jul 21	07 Jul 21
Last day	07 Jun 21	01 Jun 21	15 Jun 21	15 Jun 21	29 Jun 21	29 Jun 21	12 Jul 21	13 Jul 21
**Household size**								
1	356 (33.8%)	NA	324 (33.7%)	NA	294 (34.2%)	NA	280 (36.3%)	NA
2	400 (38.0%)	16 (6.3%)	384 (40.0%)	15 (6.8%)	366 (42.5%)	14 (7.2%)	304 (39.4%)	10 (5.7%)
3-5	291 (27.6%)	239 (93.4%)	249 (25.9%)	207 (93.2%)	200 (23.2%)	180 (92.3%)	186 (24.1%)	166 (94.3%)
6+	7 (0.7%)	1 (0.4%)	4 (0.4%)	NA	1 (0.1%)	1 (0.5%)	1 (0.1%)	NA

Note: Wave A refers to CoMix surveys collecting behavioural data for adults whereas Wave C refers to CoMix surveys collecting behavioural data from adults responding on behalf of a randomly selected child.

1.4 France

Table S4. CoMix sample characteristics for France

Characteristic	Wave A 1	Wave A 2	Wave A 3	Wave A 4	Wave A 5	Wave A 6	Wave A 7	Wave C 1	Wave C 2
**All**	1564	1320	1122	954	812	690	585	308	297
**Age**									
0-4	NA	NA	NA	NA	NA	NA	NA	47 (15.3%)	40 (13.5%)
5-11	NA	NA	NA	NA	NA	NA	NA	85 (27.6%)	80 (26.9%)
12-15	NA	NA	NA	NA	NA	NA	NA	94 (30.5%)	99 (33.3%)
16-17	NA	NA	NA	NA	NA	NA	NA	82 (26.6%)	68 (22.9%)
18-29	241 (15.4%)	195 (14.8%)	133 (11.9%)	171 (17.9%)	110 (13.5%)	105 (15.2%)	104 (17.8%)	NA	NA
30-39	265 (16.9%)	217 (16.4%)	160 (14.3%)	177 (18.6%)	147 (18.1%)	135 (19.6%)	123 (21.0%)	NA	NA
40-49	256 (16.4%)	228 (17.3%)	186 (16.6%)	164 (17.2%)	153 (18.8%)	79 (11.4%)	98 (16.8%)	NA	NA
50-59	251 (16.0%)	212 (16.1%)	190 (16.9%)	158 (16.6%)	182 (22.4%)	7 (1.0%)	79 (13.5%)	NA	NA
60-69	330 (21.1%)	284 (21.5%)	269 (24.0%)	186 (19.5%)	181 (22.3%)	212 (30.7%)	115 (19.7%)	NA	NA
70-120	221 (14.1%)	184 (13.9%)	184 (16.4%)	98 (10.3%)	39 (4.8%)	152 (22.0%)	66 (11.3%)	NA	NA
NA/Other	NA	NA	NA	NA	NA	NA	NA	NA	10 (3.4%)
**Gender**									
Female	737 (47.1%)	601 (45.5%)	539 (48.0%)	415 (43.5%)	385 (47.4%)	341 (49.4%)	255 (43.6%)	224 (72.7%)	197 (66.3%)
Male	820 (52.4%)	716 (54.2%)	581 (51.8%)	534 (56.0%)	426 (52.5%)	347 (50.3%)	328 (56.1%)	84 (27.3%)	100 (33.7%)
Other or Missing	7 (0.4%)	3 (0.2%)	2 (0.2%)	5 (0.5%)	1 (0.1%)	2 (0.3%)	2 (0.3%)	NA	NA
**Day**									
Sunday	883 (56.5%)	135 (10.2%)	NA	NA	55 (6.8%)	NA	NA	NA	17 (5.7%)
Monday	648 (41.4%)	257 (19.5%)	NA	NA	NA	NA	NA	10 (3.2%)	30 (10.1%)
Tuesday	7 (0.4%)	NA	77 (6.9%)	391 (41.0%)	93 (11.5%)	203 (29.4%)	565 (96.6%)	42 (13.6%)	2 (0.7%)
Wednesday	26 (1.7%)	41 (3.1%)	898 (80.0%)	401 (42.0%)	135 (16.6%)	204 (29.6%)	20 (3.4%)	181 (58.8%)	115 (38.7%)
Thursday	NA	735 (55.7%)	147 (13.1%)	143 (15.0%)	122 (15.0%)	178 (25.8%)	NA	38 (12.3%)	88 (29.6%)
Friday	NA	114 (8.6%)	NA	19 (2.0%)	170 (20.9%)	105 (15.2%)	NA	28 (9.1%)	33 (11.1%)
Saturday	NA	38 (2.9%)	NA	NA	237 (29.2%)	NA	NA	9 (2.9%)	12 (4.0%)
**Data collection**									
First day	21 Dec 20	07 Jan 21	20 Jan 21	17 Feb 21	03 Mar 21	17 Mar 21	14 Apr 21	03 Feb 21	02 Apr 21
Last day	29 Dec 20	12 Jan 21	22 Jan 21	20 Feb 21	08 Mar 21	20 Mar 21	15 Apr 21	09 Feb 21	08 Apr 21
**Household size**									
1	371 (23.7%)	304 (23.0%)	286 (25.5%)	232 (24.3%)	201 (24.8%)	175 (25.4%)	137 (23.4%)	NA	NA
2	595 (38.0%)	501 (38.0%)	449 (40.0%)	338 (35.4%)	283 (34.9%)	294 (42.6%)	206 (35.2%)	18 (5.8%)	21 (7.1%)
3-5	571 (36.5%)	493 (37.3%)	374 (33.3%)	368 (38.6%)	314 (38.7%)	211 (30.6%)	230 (39.3%)	282 (91.6%)	266 (89.6%)
6+	27 (1.7%)	22 (1.7%)	13 (1.2%)	16 (1.7%)	14 (1.7%)	10 (1.4%)	12 (2.1%)	7 (2.3%)	8 (2.7%)
0	NA	NA	NA	NA	NA	NA	NA	1 (0.3%)	2 (0.7%)

Note: Wave A refers to CoMix surveys collecting behavioural data for adults whereas Wave C refers to CoMix surveys collecting behavioural data from adults responding on behalf of a randomly selected child.

1.5 Italy

Table S5. CoMix sample characteristics for Italy

Characteristic	Wave A 1	Wave A 2	Wave A 3	Wave A 4	Wave A 5	Wave A 6	Wave A 7	Wave C 1	Wave C 2
**All**	1559	1324	1125	955	811	689	591	306	297
**Age**									
0-4	NA	NA	NA	NA	NA	NA	NA	84 (27.5%)	77 (25.9%)
5-11	NA	NA	NA	NA	NA	NA	NA	85 (27.8%)	86 (29.0%)
12-15	NA	NA	NA	NA	NA	NA	NA	76 (24.8%)	71 (23.9%)
16-17	NA	NA	NA	NA	NA	NA	NA	61 (19.9%)	54 (18.2%)
18-29	240 (15.4%)	184 (13.9%)	151 (13.4%)	150 (15.7%)	107 (13.2%)	145 (21.0%)	84 (14.2%)	NA	NA
30-39	206 (13.2%)	170 (12.8%)	140 (12.4%)	140 (14.7%)	100 (12.3%)	137 (19.9%)	61 (10.3%)	NA	NA
40-49	323 (20.7%)	266 (20.1%)	238 (21.2%)	243 (25.4%)	173 (21.3%)	164 (23.8%)	131 (22.2%)	NA	NA
50-59	239 (15.3%)	206 (15.6%)	178 (15.8%)	190 (19.9%)	143 (17.6%)	64 (9.3%)	87 (14.7%)	NA	NA
60-69	348 (22.3%)	310 (23.4%)	247 (22.0%)	232 (24.3%)	172 (21.2%)	19 (2.8%)	116 (19.6%)	NA	NA
70-120	203 (13.0%)	188 (14.2%)	171 (15.2%)	NA	116 (14.3%)	160 (23.2%)	112 (19.0%)	NA	NA
NA/Other	NA	NA	NA	NA	NA	NA	NA	NA	9 (3.0%)
**Gender**									
Female	745 (47.8%)	615 (46.5%)	523 (46.5%)	438 (45.9%)	384 (47.3%)	310 (45.0%)	272 (46.0%)	302 (98.7%)	292 (98.3%)
Male	810 (52.0%)	709 (53.5%)	601 (53.4%)	517 (54.1%)	426 (52.5%)	379 (55.0%)	319 (54.0%)	1 (0.3%)	4 (1.3%)
Other or Missing	4 (0.3%)	NA	1 (0.1%)	NA	1 (0.1%)	NA	NA	3 (1.0%)	1 (0.3%)
**Day**									
Sunday	734 (47.1%)	90 (6.8%)	NA	220 (23.0%)	NA	NA	50 (8.5%)	NA	9 (3.0%)
Monday	431 (27.6%)	57 (4.3%)	NA	NA	NA	NA	8 (1.4%)	9 (2.9%)	8 (2.7%)
Tuesday	233 (14.9%)	7 (0.5%)	785 (69.8%)	191 (20.0%)	57 (7.0%)	28 (4.1%)	259 (43.8%)	32 (10.5%)	79 (26.6%)
Wednesday	130 (8.3%)	622 (47.0%)	243 (21.6%)	18 (1.9%)	6 (0.7%)	154 (22.4%)	50 (8.5%)	60 (19.6%)	NA
Thursday	11 (0.7%)	407 (30.7%)	97 (8.6%)	168 (17.6%)	748 (92.2%)	182 (26.4%)	71 (12.0%)	205 (67.0%)	176 (59.3%)
Friday	12 (0.8%)	108 (8.2%)	NA	167 (17.5%)	NA	176 (25.5%)	49 (8.3%)	NA	19 (6.4%)
Saturday	8 (0.5%)	33 (2.5%)	NA	191 (20.0%)	NA	149 (21.6%)	104 (17.6%)	NA	6 (2.0%)
**Data collection**									
First day	21 Dec 20	07 Jan 21	20 Jan 21	17 Feb 21	03 Mar 21	17 Mar 21	14 Apr 21	03 Feb 21	02 Apr 21
Last day	29 Dec 20	13 Jan 21	22 Jan 21	22 Feb 21	05 Mar 21	21 Mar 21	23 Apr 21	09 Feb 21	09 Apr 21
**Household size**									
1	184 (11.8%)	167 (12.6%)	140 (12.4%)	106 (11.1%)	109 (13.4%)	88 (12.8%)	83 (14.0%)	NA	NA
2	512 (32.8%)	446 (33.7%)	385 (34.2%)	281 (29.4%)	287 (35.4%)	225 (32.7%)	193 (32.7%)	13 (4.2%)	8 (2.7%)
3-5	842 (54.0%)	696 (52.6%)	585 (52.0%)	556 (58.2%)	409 (50.4%)	370 (53.7%)	308 (52.1%)	284 (92.8%)	277 (93.3%)
6+	21 (1.3%)	15 (1.1%)	15 (1.3%)	12 (1.3%)	6 (0.7%)	6 (0.9%)	7 (1.2%)	9 (2.9%)	11 (3.7%)
0	NA	NA	NA	NA	NA	NA	NA	NA	1 (0.3%)

Note: Wave A refers to CoMix surveys collecting behavioural data for adults whereas Wave C refers to CoMix surveys collecting behavioural data from adults responding on behalf of a randomly selected child.

1.6 Spain

Table S6. CoMix sample characteristics for Spain

Characteristic	Wave A 1	Wave A 2	Wave A 3	Wave A 4	Wave A 5	Wave A 6	Wave A 7	Wave C 1	Wave C 2
**All**	1569	1319	1126	956	815	688	586	294	297
**Age**									
0-4	NA	NA	NA	NA	NA	NA	NA	58 (19.7%)	54 (18.2%)
5-11	NA	NA	NA	NA	NA	NA	NA	125 (42.5%)	121 (40.7%)
12-15	NA	NA	NA	NA	NA	NA	NA	75 (25.5%)	88 (29.6%)
16-17	NA	NA	NA	NA	NA	NA	NA	36 (12.2%)	31 (10.4%)
18-29	238 (15.2%)	197 (14.9%)	138 (12.3%)	136 (14.2%)	90 (11.0%)	124 (18.0%)	107 (18.3%)	NA	NA
30-39	260 (16.6%)	209 (15.8%)	183 (16.3%)	162 (16.9%)	110 (13.5%)	167 (24.3%)	107 (18.3%)	NA	NA
40-49	328 (20.9%)	275 (20.8%)	232 (20.6%)	219 (22.9%)	185 (22.7%)	210 (30.5%)	135 (23.0%)	NA	NA
50-59	271 (17.3%)	220 (16.7%)	190 (16.9%)	189 (19.8%)	154 (18.9%)	127 (18.5%)	101 (17.2%)	NA	NA
60-69	299 (19.1%)	264 (20.0%)	244 (21.7%)	162 (16.9%)	198 (24.3%)	60 (8.7%)	101 (17.2%)	NA	NA
70-120	173 (11.0%)	154 (11.7%)	139 (12.3%)	88 (9.2%)	78 (9.6%)	NA	35 (6.0%)	NA	NA
NA/Other	4 (0.3%)	1 (0.1%)	2 (0.2%)	2 (0.2%)	2 (0.2%)	NA	NA	NA	NA
**Gender**									
Female	760 (48.4%)	635 (48.1%)	559 (49.6%)	466 (48.7%)	428 (52.5%)	361 (52.5%)	287 (49.0%)	205 (69.7%)	193 (65.0%)
Male	805 (51.3%)	683 (51.8%)	565 (50.2%)	488 (51.0%)	385 (47.2%)	327 (47.5%)	299 (51.0%)	89 (30.3%)	104 (35.0%)
Other or Missing	4 (0.3%)	NA	1 (0.1%)	NA	1 (0.1%)	NA	NA	3 (1.0%)	1 (0.3%)
**Day**									
Sunday	858 (54.7%)	118 (8.9%)	NA	294 (30.8%)	130 (16.0%)	NA	268 (45.7%)	NA	1 (0.3%)
Monday	572 (36.5%)	62 (4.7%)	NA	402 (42.1%)	3 (0.4%)	NA	NA	NA	2 (0.7%)
Tuesday	102 (6.5%)	NA	871 (77.4%)	52 (5.4%)	82 (10.1%)	178 (25.9%)	295 (50.3%)	NA	225 (75.8%)
Wednesday	18 (1.1%)	339 (25.7%)	230 (20.4%)	10 (1.0%)	150 (18.4%)	169 (24.6%)	18 (3.1%)	274 (93.2%)	17 (5.7%)
Thursday	6 (0.4%)	543 (41.2%)	25 (2.2%)	173 (18.1%)	146 (17.9%)	203 (29.5%)	2 (0.3%)	20 (6.8%)	41 (13.8%)
Friday	6 (0.4%)	201 (15.2%)	NA	17 (1.8%)	128 (15.7%)	138 (20.1%)	1 (0.2%)	NA	8 (2.7%)
Saturday	7 (0.4%)	56 (4.2%)	NA	8 (0.8%)	176 (21.6%)	NA	2 (0.3%)	NA	3 (1.0%)
**Data collection**									
First day	21 Dec 20	07 Jan 21	20 Jan 21	17 Feb 21	03 Mar 21	17 Mar 21	14 Apr 21	01 Apr 21	06 May 21
Last day	29 Dec 20	12 Jan 21	22 Jan 21	23 Feb 21	09 Mar 21	20 Mar 21	19 Apr 21	02 Apr 21	12 May 21
**Household size**									
1	164 (10.5%)	147 (11.1%)	130 (11.5%)	111 (11.6%)	100 (12.3%)	61 (8.9%)	60 (10.2%)	NA	NA
2	521 (33.2%)	458 (34.7%)	416 (36.9%)	323 (33.8%)	295 (36.2%)	198 (28.8%)	195 (33.3%)	10 (3.4%)	6 (2.0%)
3-5	846 (53.9%)	689 (52.2%)	558 (49.6%)	502 (52.5%)	406 (49.8%)	413 (60.0%)	322 (54.9%)	278 (94.6%)	287 (96.6%)
6+	38 (2.4%)	25 (1.9%)	22 (2.0%)	20 (2.1%)	14 (1.7%)	16 (2.3%)	9 (1.5%)	6 (2.0%)	4 (1.3%)

Note: Wave A refers to CoMix surveys collecting behavioural data for adults whereas Wave C refers to CoMix surveys collecting behavioural data from adults responding on behalf of a randomly selected child.

1.7 Austria

Table S7. CoMix sample characteristics for Austria

Characteristic	Wave A 1	Wave A 2	Wave A 3	Wave A 4	Wave A 5	Wave A 6	Wave A 7	Wave C 1	Wave C 2
**All**	1554	1317	1126	955	812	687	586	306	299
**Age**									
0-4	NA	NA	NA	NA	NA	NA	NA	121 (39.5%)	105 (35.1%)
5-11	NA	NA	NA	NA	NA	NA	NA	95 (31.0%)	108 (36.1%)
12-15	NA	NA	NA	NA	NA	NA	NA	63 (20.6%)	59 (19.7%)
16-17	NA	NA	NA	NA	NA	NA	NA	27 (8.8%)	23 (7.7%)
18-29	306 (19.7%)	257 (19.5%)	189 (16.8%)	164 (17.2%)	142 (17.5%)	125 (18.2%)	122 (20.8%)	NA	NA
30-39	277 (17.8%)	237 (18.0%)	202 (17.9%)	151 (15.8%)	124 (15.3%)	122 (17.8%)	102 (17.4%)	NA	NA
40-49	259 (16.7%)	224 (17.0%)	195 (17.3%)	159 (16.6%)	127 (15.6%)	111 (16.2%)	104 (17.7%)	NA	NA
50-59	306 (19.7%)	234 (17.8%)	224 (19.9%)	197 (20.6%)	170 (20.9%)	138 (20.1%)	106 (18.1%)	NA	NA
60-69	275 (17.7%)	235 (17.8%)	213 (18.9%)	182 (19.1%)	173 (21.3%)	123 (17.9%)	98 (16.7%)	NA	NA
70-120	131 (8.4%)	130 (9.9%)	103 (9.1%)	102 (10.7%)	76 (9.4%)	68 (9.9%)	54 (9.2%)	NA	NA
NA/Other	NA	NA	NA	NA	NA	NA	NA	NA	4 (1.3%)
**Gender**									
Female	753 (48.5%)	632 (48.0%)	540 (48.0%)	434 (45.4%)	351 (43.2%)	322 (46.9%)	267 (45.6%)	202 (66.0%)	198 (66.2%)
Male	798 (51.4%)	684 (51.9%)	585 (52.0%)	519 (54.3%)	459 (56.5%)	365 (53.1%)	316 (53.9%)	101 (33.0%)	101 (33.8%)
Other or Missing	3 (0.2%)	1 (0.1%)	1 (0.1%)	2 (0.2%)	2 (0.2%)	NA	3 (0.5%)	3 (1.0%)	NA
**Day**									
Sunday	375 (24.1%)	68 (5.2%)	NA	386 (40.4%)	160 (19.7%)	447 (65.1%)	369 (63.0%)	107 (35.0%)	14 (4.7%)
Monday	367 (23.6%)	53 (4.0%)	NA	NA	NA	NA	NA	10 (3.3%)	18 (6.0%)
Tuesday	242 (15.6%)	135 (10.3%)	95 (8.4%)	41 (4.3%)	57 (7.0%)	77 (11.2%)	21 (3.6%)	21 (6.9%)	5 (1.7%)
Wednesday	112 (7.2%)	44 (3.3%)	904 (80.3%)	113 (11.8%)	122 (15.0%)	131 (19.1%)	55 (9.4%)	39 (12.7%)	153 (51.2%)
Thursday	52 (3.3%)	538 (40.9%)	104 (9.2%)	105 (11.0%)	175 (21.6%)	20 (2.9%)	41 (7.0%)	48 (15.7%)	60 (20.1%)
Friday	25 (1.6%)	303 (23.0%)	23 (2.0%)	24 (2.5%)	134 (16.5%)	4 (0.6%)	50 (8.5%)	34 (11.1%)	NA
Saturday	381 (24.5%)	176 (13.4%)	NA	286 (29.9%)	164 (20.2%)	8 (1.2%)	50 (8.5%)	47 (15.4%)	49 (16.4%)
**Data collection**									
First day	22 Dec 20	07 Jan 21	20 Jan 21	17 Feb 21	03 Mar 21	17 Mar 21	14 Apr 21	03 Feb 21	02 Apr 21
Last day	06 Jan 21	13 Jan 21	23 Jan 21	22 Feb 21	08 Mar 21	22 Mar 21	19 Apr 21	09 Feb 21	08 Apr 21
**Household size**									
1	412 (26.5%)	367 (27.9%)	311 (27.6%)	275 (28.8%)	245 (30.2%)	199 (29.0%)	168 (28.7%)	NA	NA
2	621 (40.0%)	526 (39.9%)	461 (40.9%)	389 (40.7%)	325 (40.0%)	285 (41.5%)	243 (41.5%)	24 (7.8%)	27 (9.0%)
3-5	494 (31.8%)	405 (30.8%)	337 (29.9%)	278 (29.1%)	232 (28.6%)	193 (28.1%)	167 (28.5%)	273 (89.2%)	265 (88.6%)
6+	27 (1.7%)	19 (1.4%)	17 (1.5%)	13 (1.4%)	10 (1.2%)	10 (1.5%)	8 (1.4%)	9 (2.9%)	7 (2.3%)

Note: Wave A refers to CoMix surveys collecting behavioural data for adults whereas Wave C refers to CoMix surveys collecting behavioural data from adults responding on behalf of a randomly selected child.

1.8 Denmark

Table S8. CoMix sample characteristics for Denmark

Characteristic	Wave A 1	Wave A 2	Wave A 3	Wave A 4	Wave A 5	Wave A 6	Wave A 7	Wave C 1	Wave C 2
**All**	1558	1323	1125	959	815	690	587	307	294
**Age**									
0-4	NA	NA	NA	NA	NA	NA	NA	85 (27.7%)	78 (26.5%)
5-11	NA	NA	NA	NA	NA	NA	NA	112 (36.5%)	101 (34.4%)
12-15	NA	NA	NA	NA	NA	NA	NA	90 (29.3%)	98 (33.3%)
16-17	NA	NA	NA	NA	NA	NA	NA	20 (6.5%)	14 (4.8%)
18-29	273 (17.5%)	196 (14.8%)	149 (13.2%)	124 (12.9%)	108 (13.3%)	77 (11.2%)	69 (11.8%)	NA	NA
30-39	160 (10.3%)	151 (11.4%)	119 (10.6%)	98 (10.2%)	87 (10.7%)	72 (10.4%)	59 (10.1%)	NA	NA
40-49	198 (12.7%)	201 (15.2%)	164 (14.6%)	131 (13.7%)	102 (12.5%)	92 (13.3%)	77 (13.1%)	NA	NA
50-59	316 (20.3%)	295 (22.3%)	249 (22.1%)	213 (22.2%)	200 (24.5%)	169 (24.5%)	142 (24.2%)	NA	NA
60-69	333 (21.4%)	280 (21.2%)	257 (22.8%)	223 (23.3%)	187 (22.9%)	174 (25.2%)	154 (26.2%)	NA	NA
70-120	278 (17.8%)	200 (15.1%)	187 (16.6%)	170 (17.7%)	131 (16.1%)	106 (15.4%)	86 (14.7%)	NA	NA
NA/Other	NA	NA	NA	NA	NA	NA	NA	NA	3 (1.0%)
**Gender**									
Female	768 (49.3%)	609 (46.0%)	510 (45.3%)	448 (46.7%)	396 (48.6%)	325 (47.1%)	271 (46.2%)	172 (56.0%)	168 (57.1%)
Male	788 (50.6%)	713 (53.9%)	612 (54.4%)	509 (53.1%)	416 (51.0%)	364 (52.8%)	315 (53.7%)	135 (44.0%)	125 (42.5%)
Other or Missing	2 (0.1%)	1 (0.1%)	3 (0.3%)	2 (0.2%)	3 (0.4%)	1 (0.1%)	1 (0.2%)	NA	1 (0.3%)
**Day**									
Sunday	219 (14.1%)	115 (8.7%)	NA	28 (2.9%)	55 (6.7%)	NA	NA	21 (6.8%)	13 (4.4%)
Monday	494 (31.7%)	33 (2.5%)	NA	337 (35.1%)	57 (7.0%)	NA	NA	14 (4.6%)	123 (41.8%)
Tuesday	361 (23.2%)	116 (8.8%)	156 (13.9%)	139 (14.5%)	117 (14.4%)	25 (3.6%)	84 (14.3%)	32 (10.4%)	40 (13.6%)
Wednesday	143 (9.2%)	170 (12.8%)	833 (74.0%)	190 (19.8%)	163 (20.0%)	140 (20.3%)	74 (12.6%)	72 (23.5%)	43 (14.6%)
Thursday	170 (10.9%)	738 (55.8%)	136 (12.1%)	51 (5.3%)	175 (21.5%)	202 (29.3%)	429 (73.1%)	71 (23.1%)	52 (17.7%)
Friday	112 (7.2%)	102 (7.7%)	NA	117 (12.2%)	126 (15.5%)	243 (35.2%)	NA	59 (19.2%)	8 (2.7%)
Saturday	59 (3.8%)	49 (3.7%)	NA	97 (10.1%)	122 (15.0%)	80 (11.6%)	NA	38 (12.4%)	15 (5.1%)
**Data collection**									
First day	22 Dec 20	07 Jan 21	20 Jan 21	17 Feb 21	03 Mar 21	17 Mar 21	14 Apr 21	03 Feb 21	02 Apr 21
Last day	06 Jan 21	14 Jan 21	22 Jan 21	23 Feb 21	09 Mar 21	21 Mar 21	16 Apr 21	09 Feb 21	12 Apr 21
**Household size**									
1	445 (28.6%)	380 (28.7%)	338 (30.0%)	289 (30.1%)	248 (30.4%)	218 (31.6%)	179 (30.5%)	NA	NA
2	737 (47.3%)	600 (45.4%)	511 (45.4%)	433 (45.2%)	365 (44.8%)	316 (45.8%)	269 (45.8%)	23 (7.5%)	20 (6.8%)
3-5	366 (23.5%)	336 (25.4%)	266 (23.6%)	231 (24.1%)	196 (24.0%)	155 (22.5%)	135 (23.0%)	272 (88.6%)	263 (89.5%)
6+	10 (0.6%)	7 (0.5%)	10 (0.9%)	6 (0.6%)	6 (0.7%)	1 (0.1%)	4 (0.7%)	12 (3.9%)	11 (3.7%)

Note: Wave A refers to CoMix surveys collecting behavioural data for adults whereas Wave C refers to CoMix surveys collecting behavioural data from adults responding on behalf of a randomly selected child.

1.9 Poland

Table S9. CoMix sample characteristics for Poland

Characteristic	Wave A 1	Wave A 2	Wave A 3	Wave A 4	Wave A 5	Wave A 6	Wave A 7	Wave C 1	Wave C 2
**All**	1543	1310	1125	954	809	684	586	305	292
**Age**									
0-4	NA	NA	NA	NA	NA	NA	NA	89 (29.2%)	84 (28.8%)
5-11	NA	NA	NA	NA	NA	NA	NA	104 (34.1%)	98 (33.6%)
12-15	NA	NA	NA	NA	NA	NA	NA	63 (20.7%)	62 (21.2%)
16-17	NA	NA	NA	NA	NA	NA	NA	49 (16.1%)	41 (14.0%)
18-29	317 (20.5%)	267 (20.4%)	206 (18.3%)	163 (17.1%)	149 (18.4%)	134 (19.6%)	81 (13.8%)	NA	NA
30-39	287 (18.6%)	246 (18.8%)	192 (17.1%)	195 (20.4%)	159 (19.7%)	160 (23.4%)	111 (18.9%)	NA	NA
40-49	299 (19.4%)	245 (18.7%)	206 (18.3%)	220 (23.1%)	149 (18.4%)	135 (19.7%)	90 (15.4%)	NA	NA
50-59	266 (17.2%)	236 (18.0%)	207 (18.4%)	201 (21.1%)	138 (17.1%)	103 (15.1%)	113 (19.3%)	NA	NA
60-69	284 (18.4%)	246 (18.8%)	243 (21.6%)	131 (13.7%)	173 (21.4%)	112 (16.4%)	139 (23.7%)	NA	NA
70-120	90 (5.8%)	70 (5.3%)	71 (6.3%)	44 (4.6%)	41 (5.1%)	40 (5.8%)	52 (8.9%)	NA	NA
NA/Other	NA	NA	NA	NA	NA	NA	NA	NA	7 (2.4%)
**Gender**									
Female	804 (52.1%)	747 (57.0%)	588 (52.3%)	541 (56.7%)	470 (58.1%)	404 (59.1%)	347 (59.2%)	169 (55.4%)	168 (57.5%)
Male	739 (47.9%)	563 (43.0%)	537 (47.7%)	413 (43.3%)	339 (41.9%)	280 (40.9%)	239 (40.8%)	136 (44.6%)	124 (42.5%)
**Day**									
Sunday	38 (2.5%)	46 (3.5%)	6 (0.5%)	220 (23.1%)	161 (19.9%)	NA	9 (1.5%)	52 (17.0%)	3 (1.0%)
Monday	311 (20.2%)	107 (8.2%)	8 (0.7%)	NA	NA	NA	NA	10 (3.3%)	67 (22.9%)
Tuesday	813 (52.7%)	82 (6.3%)	849 (75.5%)	130 (13.6%)	115 (14.2%)	67 (9.8%)	56 (9.6%)	80 (26.2%)	23 (7.9%)
Wednesday	234 (15.2%)	365 (27.9%)	206 (18.3%)	87 (9.1%)	25 (3.1%)	70 (10.2%)	74 (12.6%)	23 (7.5%)	145 (49.7%)
Thursday	77 (5.0%)	552 (42.1%)	38 (3.4%)	162 (17.0%)	63 (7.8%)	170 (24.9%)	66 (11.3%)	14 (4.6%)	46 (15.8%)
Friday	42 (2.7%)	125 (9.5%)	7 (0.6%)	162 (17.0%)	392 (48.5%)	169 (24.7%)	334 (57.0%)	48 (15.7%)	7 (2.4%)
Saturday	28 (1.8%)	33 (2.5%)	11 (1.0%)	193 (20.2%)	53 (6.6%)	208 (30.4%)	47 (8.0%)	78 (25.6%)	1 (0.3%)
**Data collection**									
First day	22 Dec 20	07 Jan 21	20 Jan 21	17 Feb 21	03 Mar 21	17 Mar 21	14 Apr 21	03 Feb 21	02 Apr 21
Last day	06 Jan 21	13 Jan 21	26 Jan 21	22 Feb 21	08 Mar 21	21 Mar 21	19 Apr 21	09 Feb 21	08 Apr 21
**Household size**									
1	195 (12.6%)	183 (14.0%)	159 (14.1%)	128 (13.4%)	119 (14.7%)	87 (12.7%)	94 (16.0%)	NA	NA
2	446 (28.9%)	360 (27.5%)	355 (31.6%)	252 (26.4%)	227 (28.1%)	175 (25.6%)	175 (29.9%)	7 (2.3%)	2 (0.7%)
3-5	826 (53.5%)	709 (54.1%)	564 (50.1%)	536 (56.2%)	427 (52.8%)	395 (57.7%)	298 (50.9%)	284 (93.1%)	275 (94.2%)
6+	76 (4.9%)	58 (4.4%)	47 (4.2%)	38 (4.0%)	36 (4.4%)	27 (3.9%)	19 (3.2%)	14 (4.6%)	15 (5.1%)

Note: Wave A refers to CoMix surveys collecting behavioural data for adults whereas Wave C refers to CoMix surveys collecting behavioural data from adults responding on behalf of a randomly selected child.

1.10 Portugal

Table S10. CoMix sample characteristics for Portugal

Characteristic	Wave A 1	Wave A 2	Wave A 3	Wave A 4	Wave A 5	Wave A 6	Wave A 7	Wave C 1	Wave C 2
**All**	1549	1309	1124	959	813	691	586	291	294
**Age**									
0-4	NA	NA	NA	NA	NA	NA	NA	70 (24.1%)	69 (23.5%)
5-11	NA	NA	NA	NA	NA	NA	NA	97 (33.3%)	93 (31.6%)
12-15	NA	NA	NA	NA	NA	NA	NA	72 (24.7%)	74 (25.2%)
16-17	NA	NA	NA	NA	NA	NA	NA	52 (17.9%)	46 (15.6%)
18-29	300 (19.4%)	200 (15.3%)	197 (17.5%)	156 (16.3%)	132 (16.2%)	77 (11.1%)	62 (10.6%)	NA	NA
30-39	239 (15.4%)	234 (17.9%)	157 (14.0%)	166 (17.3%)	135 (16.6%)	111 (16.1%)	92 (15.7%)	NA	NA
40-49	304 (19.6%)	313 (23.9%)	224 (19.9%)	219 (22.8%)	163 (20.0%)	172 (24.9%)	138 (23.5%)	NA	NA
50-59	304 (19.6%)	252 (19.3%)	217 (19.3%)	166 (17.3%)	160 (19.7%)	131 (19.0%)	118 (20.1%)	NA	NA
60-69	272 (17.6%)	214 (16.3%)	226 (20.1%)	175 (18.2%)	159 (19.6%)	129 (18.7%)	118 (20.1%)	NA	NA
70-120	130 (8.4%)	96 (7.3%)	103 (9.2%)	77 (8.0%)	64 (7.9%)	71 (10.3%)	58 (9.9%)	NA	NA
NA/Other	NA	NA	NA	NA	NA	NA	NA	NA	12 (4.1%)
**Gender**									
Female	715 (46.2%)	605 (46.2%)	483 (43.0%)	461 (48.1%)	369 (45.4%)	320 (46.3%)	246 (42.0%)	130 (44.7%)	138 (46.9%)
Male	829 (53.5%)	702 (53.6%)	640 (56.9%)	496 (51.7%)	442 (54.4%)	370 (53.5%)	340 (58.0%)	161 (55.3%)	156 (53.1%)
Other or Missing	5 (0.3%)	2 (0.2%)	1 (0.1%)	2 (0.2%)	2 (0.2%)	1 (0.1%)	NA	NA	NA
**Day**									
Sunday	80 (5.2%)	637 (48.7%)	NA	350 (36.5%)	504 (62.0%)	NA	262 (44.7%)	90 (30.9%)	95 (32.3%)
Monday	271 (17.5%)	164 (12.5%)	NA	142 (14.8%)	NA	NA	109 (18.6%)	60 (20.6%)	40 (13.6%)
Tuesday	527 (34.0%)	103 (7.9%)	190 (16.9%)	18 (1.9%)	33 (4.1%)	53 (7.7%)	4 (0.7%)	21 (7.2%)	3 (1.0%)
Wednesday	352 (22.7%)	309 (23.6%)	423 (37.6%)	85 (8.9%)	16 (2.0%)	240 (34.7%)	47 (8.0%)	85 (29.2%)	62 (21.1%)
Thursday	123 (7.9%)	68 (5.2%)	379 (33.7%)	106 (11.1%)	82 (10.1%)	345 (49.9%)	73 (12.5%)	35 (12.0%)	87 (29.6%)
Friday	132 (8.5%)	15 (1.1%)	92 (8.2%)	99 (10.3%)	79 (9.7%)	53 (7.7%)	44 (7.5%)	NA	4 (1.4%)
Saturday	64 (4.1%)	13 (1.0%)	40 (3.6%)	159 (16.6%)	99 (12.2%)	NA	47 (8.0%)	NA	3 (1.0%)
**Data collection**									
First day	22 Dec 20	08 Jan 21	20 Jan 21	17 Feb 21	03 Mar 21	17 Mar 21	15 Apr 21	05 Apr 21	07 May 21
Last day	07 Jan 21	14 Jan 21	24 Jan 21	23 Feb 21	08 Mar 21	20 Mar 21	22 Apr 21	09 Apr 21	14 May 21
**Household size**									
1	203 (13.1%)	179 (13.7%)	159 (14.1%)	137 (14.3%)	115 (14.1%)	94 (13.6%)	85 (14.5%)	NA	NA
2	517 (33.4%)	444 (33.9%)	409 (36.4%)	342 (35.7%)	295 (36.3%)	256 (37.0%)	200 (34.1%)	12 (4.1%)	9 (3.1%)
3-5	816 (52.7%)	675 (51.6%)	547 (48.7%)	474 (49.4%)	394 (48.5%)	334 (48.3%)	296 (50.5%)	275 (94.5%)	282 (95.9%)
6+	13 (0.8%)	11 (0.8%)	9 (0.8%)	6 (0.6%)	9 (1.1%)	7 (1.0%)	5 (0.9%)	4 (1.4%)	3 (1.0%)

Note: Wave A refers to CoMix surveys collecting behavioural data for adults whereas Wave C refers to CoMix surveys collecting behavioural data from adults responding on behalf of a randomly selected child.

1.11 Greece

Table S11. CoMix sample characteristics for Greece

Characteristic	Wave A 1	Wave A 2	Wave A 3	Wave A 4	Wave A 5	Wave A 6	Wave C 1	Wave C 2
**All**	1557	1319	1120	948	810	689	307	295
**Age**								
0-4	NA	NA	NA	NA	NA	NA	54 (17.6%)	45 (15.3%)
5-11	NA	NA	NA	NA	NA	NA	126 (41.0%)	120 (40.7%)
12-15	NA	NA	NA	NA	NA	NA	74 (24.1%)	78 (26.4%)
16-17	NA	NA	NA	NA	NA	NA	53 (17.3%)	42 (14.2%)
18-29	327 (21.0%)	221 (16.8%)	171 (15.3%)	146 (15.4%)	118 (14.6%)	86 (12.5%)	NA	NA
30-39	335 (21.5%)	313 (23.7%)	239 (21.3%)	197 (20.8%)	156 (19.3%)	132 (19.2%)	NA	NA
40-49	373 (24.0%)	350 (26.5%)	303 (27.1%)	239 (25.2%)	223 (27.5%)	192 (27.9%)	NA	NA
50-59	342 (22.0%)	293 (22.2%)	277 (24.7%)	245 (25.8%)	206 (25.4%)	189 (27.4%)	NA	NA
60-69	151 (9.7%)	122 (9.2%)	112 (10.0%)	101 (10.7%)	86 (10.6%)	75 (10.9%)	NA	NA
70-120	29 (1.9%)	20 (1.5%)	18 (1.6%)	20 (2.1%)	21 (2.6%)	15 (2.2%)	NA	NA
NA/Other	NA	NA	NA	NA	NA	NA	NA	10 (3.4%)
**Gender**								
Female	778 (50.0%)	677 (51.3%)	542 (48.4%)	458 (48.3%)	368 (45.4%)	325 (47.2%)	151 (49.2%)	145 (49.2%)
Male	774 (49.7%)	641 (48.6%)	576 (51.4%)	488 (51.5%)	438 (54.1%)	361 (52.4%)	156 (50.8%)	149 (50.5%)
Other or Missing	5 (0.3%)	1 (0.1%)	2 (0.2%)	2 (0.2%)	4 (0.5%)	3 (0.4%)	NA	1 (0.3%)
**Day**								
Sunday	247 (15.9%)	326 (24.7%)	528 (47.1%)	382 (40.3%)	223 (27.5%)	11 (1.6%)	NA	13 (4.4%)
Monday	224 (14.4%)	334 (25.3%)	122 (10.9%)	50 (5.3%)	13 (1.6%)	NA	10 (3.3%)	2 (0.7%)
Tuesday	103 (6.6%)	396 (30.0%)	34 (3.0%)	8 (0.8%)	NA	NA	NA	128 (43.4%)
Wednesday	319 (20.5%)	199 (15.1%)	58 (5.2%)	47 (5.0%)	52 (6.4%)	49 (7.1%)	NA	51 (17.3%)
Thursday	460 (29.5%)	51 (3.9%)	181 (16.2%)	196 (20.7%)	201 (24.8%)	266 (38.6%)	225 (73.3%)	64 (21.7%)
Friday	90 (5.8%)	6 (0.5%)	47 (4.2%)	157 (16.6%)	176 (21.7%)	222 (32.2%)	54 (17.6%)	26 (8.8%)
Saturday	114 (7.3%)	7 (0.5%)	150 (13.4%)	108 (11.4%)	145 (17.9%)	141 (20.5%)	18 (5.9%)	11 (3.7%)
**Data collection**								
First day	18 Feb 21	19 Mar 21	01 Apr 21	15 Apr 21	29 Apr 21	13 May 21	05 Feb 21	01 Apr 21
Last day	26 Feb 21	25 Mar 21	12 Apr 21	22 Apr 21	04 May 21	17 May 21	09 Feb 21	07 Apr 21
**Household size**								
1	244 (15.7%)	219 (16.6%)	188 (16.8%)	168 (17.7%)	144 (17.8%)	129 (18.7%)	NA	NA
2	433 (27.8%)	354 (26.8%)	303 (27.1%)	266 (28.1%)	227 (28.0%)	197 (28.6%)	9 (2.9%)	9 (3.1%)
3-5	847 (54.4%)	720 (54.6%)	606 (54.1%)	500 (52.7%)	424 (52.3%)	349 (50.7%)	289 (94.1%)	277 (93.9%)
6+	33 (2.1%)	26 (2.0%)	23 (2.1%)	14 (1.5%)	15 (1.9%)	14 (2.0%)	9 (2.9%)	9 (3.1%)

Note: Wave A refers to CoMix surveys collecting behavioural data for adults whereas Wave C refers to CoMix surveys collecting behavioural data from adults responding on behalf of a randomly selected child.

1.12 Slovenia

Table S12. CoMix sample characteristics for Slovenia

Characteristic	Wave A 1	Wave A 2	Wave A 3	Wave A 4	Wave A 5	Wave A 6	Wave C 1
**All**	1542	1315	1119	947	907	797	300
**Age**							
0-4	NA	NA	NA	NA	NA	NA	73 (24.3%)
5-11	NA	NA	NA	NA	NA	NA	108 (36.0%)
12-15	NA	NA	NA	NA	NA	NA	85 (28.3%)
16-17	NA	NA	NA	NA	NA	NA	34 (11.3%)
18-29	308 (20.0%)	225 (17.1%)	181 (16.2%)	128 (13.5%)	132 (14.6%)	98 (12.3%)	NA
30-39	344 (22.3%)	277 (21.1%)	231 (20.6%)	187 (19.7%)	172 (19.0%)	149 (18.7%)	NA
40-49	335 (21.7%)	262 (19.9%)	263 (23.5%)	193 (20.4%)	195 (21.5%)	165 (20.7%)	NA
50-59	279 (18.1%)	274 (20.8%)	224 (20.0%)	212 (22.4%)	201 (22.2%)	181 (22.7%)	NA
60-69	230 (14.9%)	232 (17.6%)	184 (16.4%)	189 (20.0%)	177 (19.5%)	168 (21.1%)	NA
70-120	46 (3.0%)	45 (3.4%)	36 (3.2%)	38 (4.0%)	30 (3.3%)	36 (4.5%)	NA
**Gender**							
Female	856 (55.5%)	769 (58.5%)	639 (57.1%)	539 (56.9%)	508 (56.0%)	446 (56.0%)	176 (58.7%)
Male	686 (44.5%)	546 (41.5%)	480 (42.9%)	408 (43.1%)	399 (44.0%)	351 (44.0%)	124 (41.3%)
**Day**							
Sunday	538 (34.9%)	347 (26.4%)	264 (23.6%)	530 (56.0%)	293 (32.3%)	134 (16.8%)	37 (12.3%)
Monday	171 (11.1%)	60 (4.6%)	346 (30.9%)	77 (8.1%)	1 (0.1%)	NA	54 (18.0%)
Tuesday	149 (9.7%)	217 (16.5%)	68 (6.1%)	13 (1.4%)	NA	NA	31 (10.3%)
Wednesday	136 (8.8%)	114 (8.7%)	33 (2.9%)	49 (5.2%)	55 (6.1%)	71 (8.9%)	7 (2.3%)
Thursday	351 (22.8%)	363 (27.6%)	296 (26.5%)	116 (12.2%)	250 (27.6%)	258 (32.4%)	117 (39.0%)
Friday	143 (9.3%)	110 (8.4%)	76 (6.8%)	65 (6.9%)	150 (16.5%)	172 (21.6%)	33 (11.0%)
Saturday	54 (3.5%)	104 (7.9%)	36 (3.2%)	97 (10.2%)	158 (17.4%)	162 (20.3%)	21 (7.0%)
**Data collection**							
First day	04 Mar 21	18 Mar 21	01 Apr 21	15 Apr 21	29 Apr 21	13 May 21	02 Apr 21
Last day	15 Mar 21	25 Mar 21	12 Apr 21	22 Apr 21	04 May 21	17 May 21	12 Apr 21
**Household size**							
1	199 (12.9%)	173 (13.2%)	149 (13.3%)	133 (14.0%)	134 (14.8%)	116 (14.6%)	NA
2	456 (29.6%)	419 (31.9%)	339 (30.3%)	313 (33.1%)	302 (33.3%)	273 (34.3%)	14 (4.7%)
3-5	835 (54.2%)	683 (51.9%)	595 (53.2%)	476 (50.3%)	443 (48.8%)	381 (47.8%)	276 (92.0%)
6+	52 (3.4%)	40 (3.0%)	36 (3.2%)	25 (2.6%)	28 (3.1%)	27 (3.4%)	10 (3.3%)

Note: Wave A refers to CoMix surveys collecting behavioural data for adults whereas Wave C refers to CoMix surveys collecting behavioural data from adults responding on behalf of a randomly selected child.

1.13 Croatia

Table S13. CoMix sample characteristics for Croatia

Characteristic	Wave A 1	Wave A 2	Wave A 3	Wave C 1
**All**	1548	1313	1181	299
**Age**				
0-4	NA	NA	NA	78 (26.1%)
5-11	NA	NA	NA	119 (39.8%)
12-15	NA	NA	NA	65 (21.7%)
16-17	NA	NA	NA	37 (12.4%)
18-29	292 (18.9%)	263 (20.0%)	207 (17.5%)	NA
30-39	239 (15.4%)	218 (16.6%)	195 (16.5%)	NA
40-49	289 (18.7%)	256 (19.5%)	234 (19.8%)	NA
50-59	253 (16.3%)	247 (18.8%)	200 (16.9%)	NA
60-69	366 (23.6%)	257 (19.6%)	268 (22.7%)	NA
70-120	109 (7.0%)	72 (5.5%)	77 (6.5%)	NA
**Gender**				
Female	812 (52.5%)	687 (52.3%)	617 (52.2%)	153 (51.2%)
Male	736 (47.5%)	626 (47.7%)	564 (47.8%)	146 (48.8%)
**Day**				
Sunday	394 (25.5%)	258 (19.6%)	26 (2.2%)	NA
Monday	81 (5.2%)	33 (2.5%)	149 (12.6%)	10 (3.3%)
Tuesday	229 (14.8%)	234 (17.8%)	425 (36.0%)	34 (11.4%)
Wednesday	263 (17.0%)	216 (16.5%)	335 (28.4%)	110 (36.8%)
Thursday	287 (18.5%)	301 (22.9%)	119 (10.1%)	145 (48.5%)
Friday	193 (12.5%)	37 (2.8%)	96 (8.1%)	NA
Saturday	101 (6.5%)	234 (17.8%)	31 (2.6%)	NA
**Data collection**				
First day	20 Apr 21	06 May 21	25 May 21	08 Jun 21
Last day	27 Apr 21	12 May 21	31 May 21	11 Jun 21
**Household size**				
1	178 (11.5%)	142 (10.8%)	133 (11.3%)	NA
2	492 (31.8%)	395 (30.1%)	360 (30.5%)	8 (2.7%)
3-5	828 (53.5%)	733 (55.8%)	653 (55.3%)	269 (90.0%)
6+	50 (3.2%)	43 (3.3%)	35 (3.0%)	22 (7.4%)

Note: Wave A refers to CoMix surveys collecting behavioural data for adults whereas Wave C refers to CoMix surveys collecting behavioural data from adults responding on behalf of a randomly selected child.

1.14 Estonia

Table S14. CoMix sample characteristics for Estonia

Characteristic	Wave A 1	Wave A 2	Wave A 3	Wave C 1
**All**	1547	1314	1111	297
**Age**				
0-4	NA	NA	NA	50 (16.8%)
5-11	NA	NA	NA	92 (31.0%)
12-15	NA	NA	NA	106 (35.7%)
16-17	NA	NA	NA	49 (16.5%)
18-29	242 (15.6%)	189 (14.4%)	166 (14.9%)	NA
30-39	270 (17.5%)	237 (18.0%)	197 (17.7%)	NA
40-49	255 (16.5%)	225 (17.1%)	182 (16.4%)	NA
50-59	286 (18.5%)	233 (17.7%)	200 (18.0%)	NA
60-69	271 (17.5%)	237 (18.0%)	201 (18.1%)	NA
70-120	223 (14.4%)	193 (14.7%)	165 (14.9%)	NA
**Gender**				
Female	854 (55.2%)	725 (55.2%)	599 (53.9%)	185 (62.3%)
Male	687 (44.4%)	584 (44.4%)	509 (45.8%)	112 (37.7%)
Other or Missing	6 (0.4%)	5 (0.4%)	3 (0.3%)	NA
**Day**				
Sunday	214 (13.8%)	416 (31.7%)	113 (10.2%)	NA
Monday	101 (6.5%)	121 (9.2%)	59 (5.3%)	61 (20.5%)
Tuesday	272 (17.6%)	31 (2.4%)	111 (10.0%)	13 (4.4%)
Wednesday	445 (28.8%)	79 (6.0%)	204 (18.4%)	2 (0.7%)
Thursday	492 (31.8%)	563 (42.8%)	403 (36.3%)	206 (69.4%)
Friday	17 (1.1%)	79 (6.0%)	138 (12.4%)	11 (3.7%)
Saturday	6 (0.4%)	25 (1.9%)	83 (7.5%)	4 (1.3%)
**Data collection**				
First day	20 Apr 21	06 May 21	25 May 21	08 Jun 21
Last day	28 Apr 21	14 May 21	08 Jun 21	15 Jun 21
**Household size**				
1	383 (24.8%)	320 (24.4%)	282 (25.4%)	NA
2	664 (42.9%)	568 (43.2%)	489 (44.0%)	22 (7.4%)
3-5	476 (30.8%)	405 (30.8%)	324 (29.2%)	256 (86.2%)
6+	24 (1.6%)	21 (1.6%)	16 (1.4%)	19 (6.4%)

Note: Wave A refers to CoMix surveys collecting behavioural data for adults whereas Wave C refers to CoMix surveys collecting behavioural data from adults responding on behalf of a randomly selected child.

1.15 Hungary

Table S15. CoMix sample characteristics for Hungary

Characteristic	Wave A 1	Wave C 1
**All**	1543	299
**Age**		
0-4	NA	92 (30.8%)
5-11	NA	106 (35.5%)
12-15	NA	63 (21.1%)
16-17	NA	38 (12.7%)
18-29	224 (14.5%)	NA
30-39	260 (16.9%)	NA
40-49	307 (19.9%)	NA
50-59	247 (16.0%)	NA
60-69	357 (23.1%)	NA
70-120	148 (9.6%)	NA
**Gender**		
Female	713 (46.2%)	183 (61.2%)
Male	822 (53.3%)	116 (38.8%)
Other or Missing	8 (0.5%)	NA
**Day**		
Sunday	57 (3.7%)	35 (11.7%)
Monday	105 (6.8%)	78 (26.1%)
Tuesday	531 (34.4%)	35 (11.7%)
Wednesday	157 (10.2%)	110 (36.8%)
Thursday	544 (35.3%)	39 (13.0%)
Friday	112 (7.3%)	2 (0.7%)
Saturday	37 (2.4%)	NA
**Data collection**		
First day	25 May 21	08 Jun 21
Last day	07 Jun 21	16 Jun 21
**Household size**		
1	247 (16.0%)	NA
2	603 (39.1%)	20 (6.7%)
3-5	663 (43.0%)	255 (85.3%)
6+	30 (1.9%)	24 (8.0%)

Note: Wave A refers to CoMix surveys collecting behavioural data for adults whereas Wave C refers to CoMix surveys collecting behavioural data from adults responding on behalf of a randomly selected child.

1.16 Malta

Table S16. CoMix sample characteristics for Malta

Characteristic	Wave A 1
**All**	1264
**Age**	
18-29	240 (19.0%)
30-39	306 (24.2%)
40-49	262 (20.7%)
50-59	193 (15.3%)
60-69	178 (14.1%)
70-120	85 (6.7%)
**Gender**	
Female	725 (57.4%)
Male	535 (42.3%)
Other or Missing	4 (0.3%)
**Day**	
Sunday	159 (12.6%)
Monday	138 (10.9%)
Tuesday	359 (28.4%)
Wednesday	339 (26.8%)
Thursday	173 (13.7%)
Friday	52 (4.1%)
Saturday	44 (3.5%)
**Data collection**	
First day	27 Apr 21
Last day	06 Jun 21
**Household size**	
1	121 (9.6%)
2	323 (25.6%)
3-5	796 (63.0%)
6+	24 (1.9%)

Note: Wave A refers to CoMix surveys collecting behavioural data for adults whereas Wave C refers to CoMix surveys collecting behavioural data from adults responding on behalf of a randomly selected child.

1.17 Slovakia

Table S17. CoMix sample characteristics for Slovakia

Characteristic	Wave A 1	Wave A 2	Wave A 3	Wave C 1
**All**	1552	1321	1119	300
**Age**				
0-4	NA	NA	NA	101 (33.7%)
5-11	NA	NA	NA	106 (35.3%)
12-15	NA	NA	NA	60 (20.0%)
16-17	NA	NA	NA	33 (11.0%)
18-29	270 (17.4%)	234 (17.7%)	173 (15.5%)	NA
30-39	317 (20.4%)	277 (21.0%)	225 (20.1%)	NA
40-49	314 (20.2%)	268 (20.3%)	231 (20.6%)	NA
50-59	250 (16.1%)	220 (16.7%)	192 (17.2%)	NA
60-69	298 (19.2%)	241 (18.2%)	222 (19.8%)	NA
70-120	103 (6.6%)	81 (6.1%)	76 (6.8%)	NA
**Gender**				
Female	752 (48.5%)	629 (47.6%)	547 (48.9%)	160 (53.3%)
Male	799 (51.5%)	692 (52.4%)	572 (51.1%)	140 (46.7%)
Other or Missing	1 (0.1%)	NA	NA	NA
**Day**				
Sunday	319 (20.6%)	216 (16.4%)	74 (6.6%)	60 (20.0%)
Monday	91 (5.9%)	68 (5.1%)	271 (24.2%)	34 (11.3%)
Tuesday	252 (16.2%)	187 (14.2%)	282 (25.2%)	30 (10.0%)
Wednesday	292 (18.8%)	41 (3.1%)	90 (8.0%)	142 (47.3%)
Thursday	375 (24.2%)	241 (18.2%)	275 (24.6%)	34 (11.3%)
Friday	86 (5.5%)	257 (19.5%)	119 (10.6%)	NA
Saturday	137 (8.8%)	311 (23.5%)	8 (0.7%)	NA
**Data collection**				
First day	20 Apr 21	06 May 21	25 May 21	08 Jun 21
Last day	27 Apr 21	12 May 21	02 Jun 21	14 Jun 21
**Household size**				
1	230 (14.8%)	187 (14.2%)	178 (15.9%)	NA
2	530 (34.1%)	457 (34.6%)	397 (35.5%)	11 (3.7%)
3-5	750 (48.3%)	643 (48.7%)	522 (46.6%)	273 (91.0%)
6+	42 (2.7%)	34 (2.6%)	22 (2.0%)	16 (5.3%)

Note: Wave A refers to CoMix surveys collecting behavioural data for adults whereas Wave C refers to CoMix surveys collecting behavioural data from adults responding on behalf of a randomly selected child.

1.18 Norway

Table S18. CoMix sample characteristics for Norway

Characteristic	Wave A 1	Wave A 2	Wave A 3	Wave A 4	Wave A 5	Wave A 6
**All**	1400	1182	1012	931	768	645
**Age**						
0-4	NA	NA	NA	NA	NA	NA
5-11	NA	NA	NA	NA	NA	NA
12-15	NA	NA	NA	NA	NA	NA
16-17	NA	NA	NA	NA	NA	NA
18-29	217 (16 %)	121 (10 %)	98 (10 %)	78 (8 %)	57 (7 %)	70 (11 %)
30-39	281 (20 %)	206 (17 %)	179 (18 %)	142 (15%)	121 (16 %)	99 (15 %)
40-49	228 (16 %)	204 (17 %)	173 (17 %)	158 (17%)	136 (18 %)	111 (17 %)
50-59	242 (17 %)	229 (19 %)	209 (21 %)	190 (20%)	156 (20 %)	139 (22 %)
60-69	251 (18 %)	229 (19 %)	197 (20 %)	216 (23%)	163 (21 %)	123 (19 %)
70-120	181 (13 %)	193 (16 %)	156 (15 %)	147 (16 %)	135 (18 %)	103 (16 %)
NA/Other	NA	NA	NA	NA	NA	NA
**Gender**						
Female	698 (50 %)	569 (48 %)	475 (47 %)	441 (47 %)	345 (45 %)	287 (45 %)
Male	702 (50 %)	610 (52 %)	534 (53 %)	487 (52 %)	421 (55 %)	357 (55 %)
Other or Missing	0	0	3(0.3 %)	3(0.3)	3(0.4 %)	2 (0.3 %)
**Day**						
Sunday	293 (21 %)	174 (15 %)	57 (6 %)	124 (13 %)	86 (11 %)	15 (2 %)
Monday	203 (15 %)	30 (3 %)	103 (10 %)	327 (35 %)	264 (34 %)	129 (20 %)
Tuesday	35 (3 %)	158 (13 %)	559 (55 %)	134 (14 %)	98 (13 %)	232 (36 %)
Wednesday	50 (4 %)	158 (13 %)	153 (15 %)	150 (16 %)	70 (9 %)	267 (41 %)
Thursday	448 (32 %)	490 (41 %)	79 (8 %)	140 (15 %)	85 (11 %)	2 (0.3 %)
Friday	267 (19 %)	119 (10 %)	36 (4 %)	31 (3 %)	67 (9 %)	0 (0 %)
Saturday	104 (7 %)	53 (4 %)	25 (2 %)	25 (3 %)	98 (13 %)	0 (0 %)
**Data collection**						
First day	24 Apr 20	19 May 21	09 Jun 21	21 Jul 21	25 Aug 21	23 Sep 21
Last day	30 Apr 21	26 May 21	16 Jun 21	27 Jul 21	02 Sep 21	30 Sep 21
**Household size**						
1	320 (23 %)	300 (25 %)	278 (27 %)	269 (29 %)	200 (26 %)	179 (28 %)
2	453 (32 %)	458 (39 %)	387 (38 %)	358 (38 %)	315 (41 %)	251 (39 %)
3-5	586 (42 %)	398 (34 %)	328 (32 %)	297 (32 %)	243 (32 %)	207 (32 %)
6	41 (3 %)	26 (2 %)	19 (2 %)	7 (0.8 %)	10 (1.3 %)	8 (1.2 %)

Note: Wave A refers to CoMix surveys collecting behavioural data for adults whereas Wave C refers to CoMix surveys collecting behavioural data from adults responding on behalf of a randomly selected child.

1.19 Germany

Table S19. CoMix sample characteristics for Germany.

Characteristic	Wave 1	Wave 2	Wave 3	Wave 4	Wave 5	Wave 6	Wave 7	Wave 8	Wave 9	Wave 10
**All**	1560	1356	1081	1890	1615	1496	1207	1022	869	739
**Age**										
Under 1	9 (0.6%)	4 (0.3%)	2 (0.2%)	8 (0.4%)	5 (0.3%)	3 (0.2%)	4 (0.3%)	2 (0.2%)	1 (0.1%)	NA
1-4	37 (2.4%)	32 (2.4%)	19 (1.8%)	48 (2.5%)	33 (2.0%)	24 (1.6%)	26 (2.2%)	15 (1.5%)	21 (2.4%)	13 (1.8%)
5-9	48 (3.1%)	41 (3.0%)	30 (2.8%)	62 (3.3%)	50 (3.1%)	60 (4.0%)	45 (3.7%)	36 (3.5%)	32 (3.7%)	15 (2.0%)
10-14	73 (4.7%)	63 (4.6%)	45 (4.2%)	87 (4.6%)	74 (4.6%)	77 (5.1%)	62 (5.1%)	45 (4.4%)	47 (5.4%)	38 (5.1%)
15-19	95 (6.1%)	66 (4.9%)	45 (4.2%)	108 (5.7%)	85 (5.3%)	84 (5.6%)	62 (5.1%)	57 (5.6%)	45 (5.2%)	37 (5.0%)
20-24	83 (5.3%)	60 (4.4%)	28 (2.6%)	109 (5.8%)	80 (5.0%)	68 (4.5%)	65 (5.4%)	49 (4.8%)	47 (5.4%)	33 (4.5%)
25-34	173 (11.1%)	148 (10.9%)	96 (8.9%)	219 (11.6%)	190 (11.8%)	181 (12.1%)	161 (13.3%)	135 (13.2%)	126 (14.5%)	92 (12.4%)
35-44	137 (8.8%)	124 (9.1%)	91 (8.4%)	164 (8.7%)	145 (9.0%)	135 (9.0%)	127 (10.5%)	99 (9.7%)	84 (9.7%)	66 (8.9%)
45-54	235 (15.1%)	209 (15.4%)	174 (16.1%)	275 (14.6%)	227 (14.1%)	229 (15.3%)	215 (17.8%)	166 (16.2%)	102 (11.7%)	104 (14.1%)
55-64	265 (17.0%)	244 (18.0%)	237 (21.9%)	321 (17.0%)	278 (17.2%)	258 (17.2%)	213 (17.6%)	176 (17.2%)	129 (14.8%)	103 (13.9%)
65-69	270 (17.3%)	245 (18.1%)	199 (18.4%)	313 (16.6%)	297 (18.4%)	264 (17.6%)	186 (15.4%)	199 (19.5%)	145 (16.7%)	140 (18.9%)
70-74	89 (5.7%)	73 (5.4%)	79 (7.3%)	118 (6.2%)	100 (6.2%)	79 (5.3%)	37 (3.1%)	29 (2.8%)	65 (7.5%)	65 (8.8%)
75-79	35 (2.2%)	34 (2.5%)	29 (2.7%)	48 (2.5%)	39 (2.4%)	26 (1.7%)	1 (0.1%)	9 (0.9%)	18 (2.1%)	25 (3.4%)
80-84	6 (0.4%)	5 (0.4%)	3 (0.3%)	5 (0.3%)	5 (0.3%)	2 (0.1%)	NA	2 (0.2%)	4 (0.5%)	3 (0.4%)
85+	5 (0.3%)	6 (0.4%)	4 (0.4%)	5 (0.3%)	5 (0.3%)	3 (0.2%)	NA	NA	3 (0.3%)	5 (0.7%)
Prefer not to answer	NA	2 (0.1%)	NA	NA	1 (0.1%)	2 (0.1%)	3 (0.2%)	3 (0.3%)	NA	NA
Don't know	NA	NA	NA	NA	1 (0.1%)	1 (0.1%)	NA	NA	NA	NA
**Gender**										
Female	748 (47.9%)	638 (47.1%)	536 (49.6%)	901 (47.7%)	733 (45.4%)	695 (46.5%)	565 (46.8%)	477 (46.7%)	387 (44.5%)	315 (42.6%)
Male	806 (51.7%)	717 (52.9%)	544 (50.3%)	985 (52.1%)	877 (54.3%)	797 (53.3%)	637 (52.8%)	544 (53.2%)	481 (55.4%)	424 (57.4%)
In another way	1 (0.1%)	1 (0.1%)	NA	3 (0.2%)	2 (0.1%)	2 (0.1%)	NA	NA	1 (0.1%)	NA
Prefer not to answer	3 (0.2%)	NA	1 (0.1%)	1 (0.1%)	1 (0.1%)	2 (0.1%)	5 (0.4%)	1 (0.1%)	NA	NA
Don't know	2 (0.1%)	NA	NA	NA	2 (0.1%)	NA	NA	NA	NA	NA
**Day**										
Sunday	88 (5.6%)	63 (4.6%)	66 (6.1%)	81 (4.3%)	179 (11.1%)	76 (5.1%)	108 (8.9%)	95 (9.3%)	81 (9.3%)	NA
Monday	247 (15.8%)	121 (8.9%)	36 (3.3%)	233 (12.3%)	366 (22.7%)	173 (11.6%)	153 (12.7%)	139 (13.6%)	139 (16.0%)	6 (0.8%)
Tuesday	50 (3.2%)	60 (4.4%)	128 (11.8%)	642 (34.0%)	321 (19.9%)	267 (17.8%)	139 (11.5%)	84 (8.2%)	269 (31.0%)	NA
Wednesday	63 (4.0%)	89 (6.6%)	143 (13.2%)	246 (13.0%)	NA	55 (3.7%)	241 (20.0%)	NA	120 (13.8%)	31 (4.2%)
Thursday	54 (3.5%)	293 (21.6%)	87 (8.0%)	172 (9.1%)	94 (5.8%)	446 (29.8%)	NA	NA	NA	375 (50.7%)
Friday	914 (58.6%)	613 (45.2%)	489 (45.2%)	320 (16.9%)	410 (25.4%)	354 (23.7%)	388 (32.1%)	519 (50.8%)	122 (14.0%)	327 (44.2%)
Saturday	144 (9.2%)	117 (8.6%)	132 (12.2%)	196 (10.4%)	245 (15.2%)	125 (8.4%)	178 (14.7%)	185 (18.1%)	138 (15.9%)	NA
**Data collection**										
First day	30 Apr 20	14 May 20	28 May 20	11 Jun 20	26 Jun 20	09 Jul 20	24 Jul 20	07 Aug 20	04 Sep 20	30 Sep 20
Last day	06 May 20	21 May 20	04 Jun 20	22 Jun 20	01 Jul 20	16 Jul 20	29 Jul 20	11 Aug 20	09 Sep 20	05 Oct 20
**Household size**										
1	232 (14.9%)	256 (18.9%)	268 (24.8%)	487 (25.8%)	374 (23.2%)	306 (20.5%)	277 (22.9%)	247 (24.2%)	194 (22.3%)	206 (27.9%)
2	412 (26.4%)	351 (25.9%)	270 (25.0%)	439 (23.2%)	407 (25.2%)	414 (27.7%)	332 (27.5%)	294 (28.8%)	236 (27.2%)	212 (28.7%)
3-5	863 (55.3%)	710 (52.4%)	521 (48.2%)	910 (48.1%)	803 (49.7%)	683 (45.7%)	534 (44.2%)	421 (41.2%)	388 (44.6%)	285 (38.6%)
6+	53 (3.4%)	39 (2.9%)	22 (2.0%)	54 (2.9%)	31 (1.9%)	93 (6.2%)	64 (5.3%)	60 (5.9%)	51 (5.9%)	36 (4.9%)

Table S19 (continued) CoMix sample characteristics for Germany.

Characteristic	Wave 11	Wave 12	Wave 13	Wave 14	Wave 15	Wave 16	Wave 17	Wave 18	Wave 19	Wave 20
**All**	1499	1500	1500	1490	1500	1499	997	1499	1498	1493
**Age**										
Under 1	1 (0.1%)	3 (0.2%)	5 (0.3%)	6 (0.4%)	7 (0.5%)	5 (0.3%)	4 (0.4%)	3 (0.2%)	5 (0.3%)	6 (0.4%)
1-4	25 (1.7%)	29 (1.9%)	30 (2.0%)	36 (2.4%)	25 (1.7%)	31 (2.1%)	21 (2.1%)	33 (2.2%)	36 (2.4%)	35 (2.3%)
5-9	47 (3.1%)	47 (3.1%)	56 (3.7%)	54 (3.6%)	48 (3.2%)	51 (3.4%)	34 (3.4%)	53 (3.5%)	50 (3.3%)	46 (3.1%)
10-14	67 (4.5%)	69 (4.6%)	78 (5.2%)	69 (4.6%)	75 (5.0%)	61 (4.1%)	52 (5.2%)	71 (4.7%)	71 (4.7%)	64 (4.3%)
15-19	81 (5.4%)	82 (5.5%)	70 (4.7%)	80 (5.4%)	84 (5.6%)	86 (5.7%)	54 (5.4%)	87 (5.8%)	83 (5.5%)	86 (5.8%)
20-24	79 (5.3%)	79 (5.3%)	79 (5.3%)	99 (6.6%)	99 (6.6%)	99 (6.6%)	65 (6.5%)	82 (5.5%)	101 (6.7%)	95 (6.4%)
25-34	152 (10.1%)	156 (10.4%)	155 (10.3%)	173 (11.6%)	181 (12.1%)	186 (12.4%)	126 (12.6%)	168 (11.2%)	186 (12.4%)	182 (12.2%)
35-44	136 (9.1%)	131 (8.7%)	144 (9.6%)	138 (9.3%)	145 (9.7%)	147 (9.8%)	98 (9.8%)	160 (10.7%)	143 (9.5%)	151 (10.1%)
45-54	206 (13.7%)	204 (13.6%)	211 (14.1%)	211 (14.2%)	212 (14.1%)	210 (14.0%)	135 (13.5%)	204 (13.6%)	204 (13.6%)	209 (14.0%)
55-64	265 (17.7%)	242 (16.1%)	227 (15.1%)	245 (16.4%)	242 (16.1%)	243 (16.2%)	160 (16.0%)	248 (16.5%)	240 (16.0%)	240 (16.1%)
65-69	282 (18.8%)	292 (19.5%)	274 (18.3%)	257 (17.2%)	236 (15.7%)	233 (15.5%)	142 (14.2%)	246 (16.4%)	228 (15.2%)	223 (14.9%)
70-74	109 (7.3%)	112 (7.5%)	115 (7.7%)	85 (5.7%)	96 (6.4%)	100 (6.7%)	85 (8.5%)	106 (7.1%)	99 (6.6%)	116 (7.8%)
75-79	36 (2.4%)	43 (2.9%)	43 (2.9%)	29 (1.9%)	37 (2.5%)	35 (2.3%)	16 (1.6%)	30 (2.0%)	41 (2.7%)	33 (2.2%)
80-84	3 (0.2%)	3 (0.2%)	4 (0.3%)	2 (0.1%)	4 (0.3%)	4 (0.3%)	2 (0.2%)	3 (0.2%)	5 (0.3%)	4 (0.3%)
85+	8 (0.5%)	7 (0.5%)	8 (0.5%)	5 (0.3%)	6 (0.4%)	6 (0.4%)	2 (0.2%)	4 (0.3%)	5 (0.3%)	2 (0.1%)
Prefer not to answer	2 (0.1%)	1 (0.1%)	1 (0.1%)	1 (0.1%)	3 (0.2%)	1 (0.1%)	1 (0.1%)	1 (0.1%)	1 (0.1%)	1 (0.1%)
Don't know	NA	NA	NA	NA	NA	1 (0.1%)	NA	NA	NA	NA
**Gender**										
Female	689 (46.0%)	690 (46.0%)	711 (47.4%)	701 (47.0%)	699 (46.6%)	708 (47.2%)	462 (46.3%)	711 (47.4%)	705 (47.1%)	714 (47.8%)
Male	806 (53.8%)	808 (53.9%)	786 (52.4%)	789 (53.0%)	797 (53.1%)	787 (52.5%)	533 (53.5%)	786 (52.4%)	790 (52.7%)	776 (52.0%)
In another way	2 (0.1%)	1 (0.1%)	3 (0.2%)	NA	2 (0.1%)	2 (0.1%)	1 (0.1%)	1 (0.1%)	2 (0.1%)	1 (0.1%)
Prefer not to answer	2 (0.1%)	1 (0.1%)	NA	NA	2 (0.1%)	1 (0.1%)	1 (0.1%)	NA	1 (0.1%)	2 (0.1%)
Don't know	NA	NA	NA	NA	NA	1 (0.1%)	NA	1 (0.1%)	NA	NA
**Day**										
Sunday	69 (4.6%)	87 (5.8%)	72 (4.8%)	88 (5.9%)	119 (7.9%)	79 (5.3%)	12 (1.2%)	139 (9.3%)	224 (15.0%)	117 (7.8%)
Monday	134 (8.9%)	74 (4.9%)	152 (10.1%)	148 (9.9%)	92 (6.1%)	38 (2.5%)	NA	298 (19.9%)	66 (4.4%)	303 (20.3%)
Tuesday	109 (7.3%)	4 (0.3%)	46 (3.1%)	NA	55 (3.7%)	22 (1.5%)	11 (1.1%)	174 (11.6%)	13 (0.9%)	113 (7.6%)
Wednesday	41 (2.7%)	NA	NA	195 (13.1%)	415 (27.7%)	779 (52.0%)	NA	261 (17.4%)	553 (36.9%)	175 (11.7%)
Thursday	526 (35.1%)	306 (20.4%)	670 (44.7%)	481 (32.3%)	357 (23.8%)	248 (16.5%)	479 (48.0%)	254 (16.9%)	247 (16.5%)	324 (21.7%)
Friday	501 (33.4%)	749 (49.9%)	430 (28.7%)	467 (31.3%)	226 (15.1%)	154 (10.3%)	435 (43.6%)	237 (15.8%)	238 (15.9%)	254 (17.0%)
Saturday	119 (7.9%)	280 (18.7%)	130 (8.7%)	111 (7.4%)	236 (15.7%)	179 (11.9%)	60 (6.0%)	136 (9.1%)	157 (10.5%)	207 (13.9%)
**Data collection**										
First day	14 Oct 20	29 Oct 20	05 Nov 20	25 Nov 20	09 Dec 20	23 Dec 20	28 Jan 21	24 Feb 21	17 Mar 21	07 Apr 21
Last day	21 Oct 20	03 Nov 20	10 Nov 20	30 Nov 20	15 Dec 20	30 Dec 20	02 Feb 21	03 Mar 21	26 Mar 21	15 Apr 21
**Household size**										
1	385 (25.7%)	371 (24.7%)	382 (25.5%)	405 (27.2%)	420 (28.0%)	424 (28.3%)	283 (28.4%)	420 (28.0%)	419 (28.0%)	422 (28.3%)
2	419 (28.0%)	443 (29.5%)	438 (29.2%)	429 (28.8%)	439 (29.3%)	456 (30.4%)	303 (30.4%)	465 (31.0%)	453 (30.2%)	458 (30.7%)
3-5	627 (41.8%)	628 (41.9%)	622 (41.5%)	606 (40.7%)	599 (39.9%)	573 (38.2%)	379 (38.0%)	572 (38.2%)	585 (39.1%)	576 (38.6%)
6+	68 (4.5%)	58 (3.9%)	58 (3.9%)	50 (3.4%)	42 (2.8%)	46 (3.1%)	32 (3.2%)	42 (2.8%)	41 (2.7%)	37 (2.5%)

Table S19 (continued) CoMix sample characteristics for Germany.

Characteristic	Wave 21	Wave 22
**All**	2468	2441
**Age**		
Under 1	19 (0.8%)	17 (0.7%)
1-4	74 (3.0%)	76 (3.1%)
5-9	75 (3.0%)	69 (2.8%)
10-14	112 (4.5%)	100 (4.1%)
15-19	147 (6.0%)	129 (5.3%)
20-24	152 (6.2%)	120 (4.9%)
25-34	279 (11.3%)	289 (11.8%)
35-44	234 (9.5%)	244 (10.0%)
45-54	353 (14.3%)	360 (14.7%)
55-64	396 (16.0%)	398 (16.3%)
65-69	382 (15.5%)	370 (15.2%)
70-74	174 (7.1%)	180 (7.4%)
75-79	52 (2.1%)	68 (2.8%)
80-84	9 (0.4%)	12 (0.5%)
85+	10 (0.4%)	9 (0.4%)
Prefer not to answer	NA	NA
Don't know	NA	NA
**Gender**		
Female	1165 (47.2%)	1165 (47.7%)
Male	1295 (52.5%)	1271 (52.1%)
In another way	5 (0.2%)	4 (0.2%)
Prefer not to answer	3 (0.1%)	1 (0.0%)
Don't know	NA	NA
**Day**		
Sunday	52 (2.1%)	552 (22.6%)
Monday	327 (13.2%)	356 (14.6%)
Tuesday	533 (21.6%)	167 (6.8%)
Wednesday	638 (25.9%)	356 (14.6%)
Thursday	595 (24.1%)	483 (19.8%)
Friday	286 (11.6%)	204 (8.4%)
Saturday	37 (1.5%)	323 (13.2%)
**Data collection**		
First day	12 May 21	26 May 21
Last day	24 May 21	03 Jun 21
**Household size**		
1	735 (29.8%)	745 (30.5%)
2	829 (33.6%)	864 (35.4%)
3-5	859 (34.8%)	797 (32.7%)
6+	45 (1.8%)	35 (1.4%)
