# Supplementary material for: SOCRATES-CoMix: a platform for timely and open-source contact mixing data during and in between COVID-19 surges and interventions in over 20 European countries
Source: BMC Med. 2021 Sep 29;19:254. doi: 10.1186/s12916-021-02133-y (PMC8478607; doi:10.1186/s12916-021-02133-y)
Supplement: Supplementary file 2 — Additional file 2: Country-level ethics details. Additional file 2 provides country-level details of the ethical approvals, or waivers, for the CoMix study protocol and questionnaires. Table S20. - Country-level details of the ethical approvals, or waivers, for the CoMix study protocol and questionnaires. [file 12916_2021_2133_MOESM2_ESM.pdf]

Additional file 2 for: "SOCRATES-CoMix: A platform for timely and open-source contact mixing data during and in between COVID-19 surges and interventions in over 20 European countries"

Frederik Verelst[1], Lisa Hermans[2*], Sarah Vercruysse[2], Amy Gimma[3], Pietro Coletti[2], Jantien A Backer[4], Kerry LM Wong[3], James Wambua[2], Kevin van Zandvoort[3], Lander Willem[1], Laurens Bogaardt[4], Christel Faes[2], Christopher I Jarvis[3], Jacco Wallinga[4,5], W John Edmunds[3], Philippe Beutels[1,6], Niel Hens[1,2]

1 Centre for Health Economics Research and Modelling Infectious Diseases, Vaccine and Infectious Disease Institute, University of Antwerp, Antwerp, Belgium

2 Data Science Institute and I-BioStat, Hasselt University, Hasselt, Belgium

3 London School of Hygiene and Tropical Medicine, London, UK

4 Centre for Infectious Disease Control, National Institute for Public Health and the Environment, Bilthoven, the Netherlands

5 Dept Biomedical Data Sciences, Leiden University Medical Center, Leiden, the Netherlands

6 School of Public Health and Community Medicine, The University of New South Wales, Sydney, Australia

*Corresponding author: Lisa Hermans, lisa.hermans@uhasselt.be

1 Additional file 2: Country-level ethics details.

This additional file provides country-level details of the ethical approvals, or waivers, for the CoMix study protocol and questionnaires.

Table S20 Country-level details of the ethical approvals, or waivers, for the CoMix study protocol and questionnaires.

Country	Coordinating organization(s)	Ethical Committee	Decision	Date	Reference
Belgium	University of Antwerp & Hasselt University	Ethical Committee of the University Hospital Antwerp	Approved	06/04/2020	20/13/147
The Netherlands	RIVM	Medische Ethische Toetsingscommissie Utrecht (METC Utrecht)	Waived (Medical Scientific Research Act not applicable)	20/03/2020	not applicable
UK	LSHTM	Observational / Interventions Research Ethics Committee	Approved	18/03/2020	21795
Austria	Austrian National Public Health Institute	Ethikkommission der Stadt Wien	Waived (Drugs Act, Medical Devices Act and Vienna Hospital Act not applicable)	15/06/2020	MA 15- EK/20-149-VK_NZ
Denmark	Aarhus University	The Central Denmark Region Committees on HealthResearch Ethics	Waived (not considered as health research study)	30/06/2020	Request 175 / 2020
France	Centre Hospitalier Universitaire de Poitiers	DCRI Centre Hospitalier Universitaire de Poitiers	Waived (not considered a 'recherche impliquant la personne humaine telles que définies par l'article R1121-1 du Code de la Santé Publique').	20/08/2020	not applicable
Poland	National Institute of Public Health - National Institute of Hygiene	Komisja Bioetyczna Narodowego Instytutu Zdrowia Publicznego Panstwowego Zaldadu Higieny, Warszawa	Approved	26/01/2021	Opinia nr 5/2020 z dnia 06.08.2020 r.
Portugal	Instituto Nacional de Saude Doutor Ricardo Jorge (INSA)	Comissão de Ética para a Saúde do Instituto Nacional de Saúde Doutor Ricardo Jorge	Approved	23/06/2020	not applicable
Spain	Institut de Investigació en Ciències de la Salut Germans Trias I Pujol	Comitè d'Ètica de la Investigación del Hospital Universitari Germans Trias i Pujol	Approved	04/09/2020	PI-20-242
Italy	ISI Foundation	Comitato di Bioetica d'Ateneo, Università degli studi di Torino	Approved	20/10/2020	d65925
Finland	Finnish Institute for Health and Welfare (THL)	Institutional Review Board of the Finnish Institute for Health and Welfare	Waived (Medical Research Act not applicable)	18/12/2020	not applicable
Switzerland	University of Bern	Gesundheits-, Sozial- und Integrationsdirektion Kantonale Ethikkommission für die Forschung	Approved	21/12/2020	2020-02926
Lithuania	Vilnius University	LITHUANIAN BIOETHICS COMMITTEE	Waived (Law on Ethics of Biomedical Research of the Republic of Lithuania not applicable)	20-11-2020	No. 6B-20-298/1
Greece	University of West Attica (UniWA)	Research Ethics Committee (E.H.D.E.)University of West Attica (PADA)	Approved	25/01/2021	4857 / 21-01-2021
Slovenia	National Institute of Public Health	Komisija Republike Slovenije za medicinsko etiko (KME RS)	Approved	29/12/2020	0120-534/2020/14
Croatia	Croatian Institute of Public Health (CIPH)	Etiko povjerenstvo Hrvatskog zavoda za javno zdravstvo (HZJZ)	Approved	16/02/2021	Ur. broj: 381-15-21-2
Estonia	National Institute for Health Development	Research Ethics Committee of the National Institute for Health Development	Approved	02/03/2021	673

Table S20 (continued) Country-level details of the ethical approvals, or waivers, for the CoMix study protocol and questionnaires.

Country	Coordinating organization(s)	Ethical Committee	Decision	Date	Reference
Hungary	University of Szeged	Health Science Council Scientific and Research Ethics Committee (ETT TUKEB)	Approved	21/04/2021	IV/3075- 1 /2021/EKU
Malta	Ministry for Health	HEALTH ETHICS COMMITTEE, Directorate for Health Information & Research	Approved	09/02/2021	HEC02/2021
Slovakia	Comenius University in Bratislava	Eticka komisia Univerzita Komenskeho v Bratislave Jesseniova lekarska fakulta v Martine	Approved	18/05/2020	EK 2/2021
Norway	University of Bergen	REK Regionale Komiteer For Medisinsk Og Helsefaglig Forsningsetikk	Approved	02/04/2020	128391
Germany	Universität Münster	Ethik-Kommission der Ärztekammer Westfalen-Lippe und der Westfälischen Wilhelms-Universität	Approved	2020-473-f-S	2020-473-f-S
